# Energy-driven K-means-based LEACH routing protocol for enhanced lifetime in wireless sensor networks

**DOI:** 10.1038/s41598-025-32141-4

**Published:** 2026-01-14

**Authors:** Imane Aly Saroit, Dina Tarek

**Affiliations:** Information Technology Department, Faculty of Computers and Artificial Intelligence, Cairo University, Giza, Egypt

**Keywords:** Wireless sensor networks (WSNs), Energy efficiency, Network lifetime, K-means, LEACH, DEEC, Engineering, Mathematics and computing

## Abstract

Wireless Sensor Networks (WSNs) are widely deployed in monitoring and automation applications; however, their performance is constrained by the limited battery capacity of sensor nodes. Traditional K-means-based LEACH routing protocol improves cluster formation using distance-based clustering, but it still relies on Euclidean distance, which does not accurately represent the communication energy cost. The main innovation of this paper is the introduction of an Energy-Driven K-Means-Based LEACH routing protocol in which Euclidean distance is replaced with a novel energy-proxy metric derived from the Radio Energy Dissipation Model. This allows the clustering process to favor low-energy-cost links and penalize long-range transmissions, improving cluster-head placement without increasing computational complexity. The proposed protocol is evaluated against both the traditional K-means-Based LEACH and DEEC-KM; a heterogeneous energy-aware clustering scheme. Simulation results on 100 randomly distributed nodes across deployment areas ranging from 100 × 100 m^2^ to 500 × 500 m^2^ show that the proposed approach achieves up to 16.98% improvement in network lifetime compared to K-means-Based LEACH, and consistently outperforms DEEC-KM across all densities and metrics, including lifetime, stability periods, delivered packets, and overall energy consumption. These gains are most evident in medium- and low-density networks, while maintaining comparable performance to K-means-Based LEACH in very dense deployments. Overall, the proposed protocol contributes a lightweight yet energy-aware enhancement to centralized LEACH clustering, improving scalability and extending network lifetime under energy-constrained WSN conditions.

## Introduction

 Wireless Sensor Networks (WSNs)^1–3^ are composed of spatially distributed, resource-constrained sensor nodes that cooperatively sense and transmit environmental data; such as temperature, humidity, pressure, motion, or light; to a central Base Station (BS) for processing. Due to their flexibility and low-cost deployment, WSNs have become a foundational technology in diverse domains including environmental monitoring, precision agriculture, healthcare, military surveillance, smart cities, and large-scale Internet of Things (IoT) infrastructures. However, the limited battery capacity of sensor nodes makes energy efficiency the dominant design constraint; once nodes exhaust their energy, they permanently fail, potentially causing network partitioning or complete loss of sensing coverage.

Routing thus plays a critical role in WSN performance^4,5^. A routing protocol in WSN must efficiently deliver sensed data to the BS while operating under severe limitations in computation, memory, communication range, and energy. It must also remain robust to node failures, topology changes, and heterogeneous traffic. Consequently, energy-aware, self-adaptive routing strategies are essential to extend network lifetime while preserving scalability, reliability, and Quality of Service.

Among existing solutions, the Low-Energy Adaptive Clustering Hierarchy (LEACH) protocol^6–8^ is one of the most influential hierarchical distributed routing schemes for WSNs. LEACH forms clusters to reduce long-range transmissions and periodically rotates Cluster Head (CH) roles among nodes to balance energy consumption then extends the network lifetime. LEACH-C^9^ later introduced a centralized cluster-formation model where the BS; instead of the nodes themselves; selects CHs and assigns cluster members to optimize energy usage.

K-Means-Based LEACH^10^ builds upon LEACH-C; by employing the K-means clustering algorithm at the BS to form more balanced clusters based on spatial proximity, thereby minimizing intra-cluster transmission distances. K-means^11,12^ is chosen for centralized cluster formation because LEACH-C already requires global node position and residual-energy information at the BS; therefore, performing clustering at the BS avoids on-node training or heavy local computation. K-means is deterministic, requires no training data, converges quickly, and integrates naturally with the centralized scheduling of LEACH-C. However, even though K-means-based LEACH improves cluster compactness, its reliance on pure Euclidean distance means that cluster formation remains disconnected from the actual energy dissipation characteristics of wireless communication. This mismatch creates an opportunity for innovation: enabling clustering decisions that explicitly reflect the nonlinear energy cost of transmission.

Beyond K-means-based LEACH, other clustering approaches such as DEEC-KM^13^ attempt to enhance network lifetime by selecting Cluster Heads (CHs) based primarily on residual energy. While such schemes can improve CH fairness, they still neglect the strong dependence of communication cost on transmitter–receiver distance, and therefore may form energy-imbalanced clusters, particularly in heterogeneous or sparsely distributed networks. DEEC-KM thus represents a useful baseline for comparison, but its lack of distance-awareness limits its efficiency in many topologies.

This work introduces an enhanced Energy-Driven K-Means-Based LEACH protocol. Rather than clustering purely on Euclidean distance, we propose a novel energy-proxy distance function derived from the Radio Energy Dissipation Model^9,14^. This is the key innovation of the paper: it embeds the physical-layer energy model directly into the clustering stage, guiding CH selection toward energy-optimal configurations without modifying the underlying LEACH-C architecture. While recent machine-learning based clustering can yield superior cluster-head selection in heterogeneous or highly dynamic environments, they require model training, model distribution, or additional runtime inference overhead; in contrast our energy-proxy is a light-weight, analytically motivated modification that preserves K-means’ simplicity while better aligning clusters with communication energy cost.

The proposed approach penalizes long transmitter–receiver distances more aggressively than short ones, thereby reducing overall communication cost while preserving the simplicity, determinism, and fast convergence of K-means. Unlike the traditional K-means-based LEACH, which relies purely on Euclidean distances during cluster formation and therefore does not accurately reflect the nonlinear growth of transmission energy with distance, the proposed method embeds an energy-proxy metric that enables more energy-aware clustering without increasing algorithmic complexity. Compared with DEEC-KM, which selects CHs solely based on residual energy, the proposed method provides a more balanced and energy-efficient clustering structure that better aligns with transmission energy requirements. By integrating the energy-proxy metric into K-means, the proposed method maintains the simplicity of classical clustering while introducing a new energy-sensitive behavior not present in existing LEACH variants or in DEEC-KM’s purely energy-based CH selection.

The remainder of the paper is structured as follows: “Literature review” introduces WSNs and presents related work on LEACH and energy-aware clustering in WSNs. “Proposed protocol” reviews K-Means-Based LEACH and details the proposed Energy-Driven enhancement. “Results and analysis” describes the evaluation setup and analyzes the simulation results. “Conclusion and future works” concludes the paper and outlines future research directions.

## Literature review

This section introduces WSNs, followed by a discussion of the LEACH routing protocol and its various adaptations, and finally some energy-efficient non-LEACH routing protocols were mentioned.

### Wireless sensor network

**Fig. 1:**
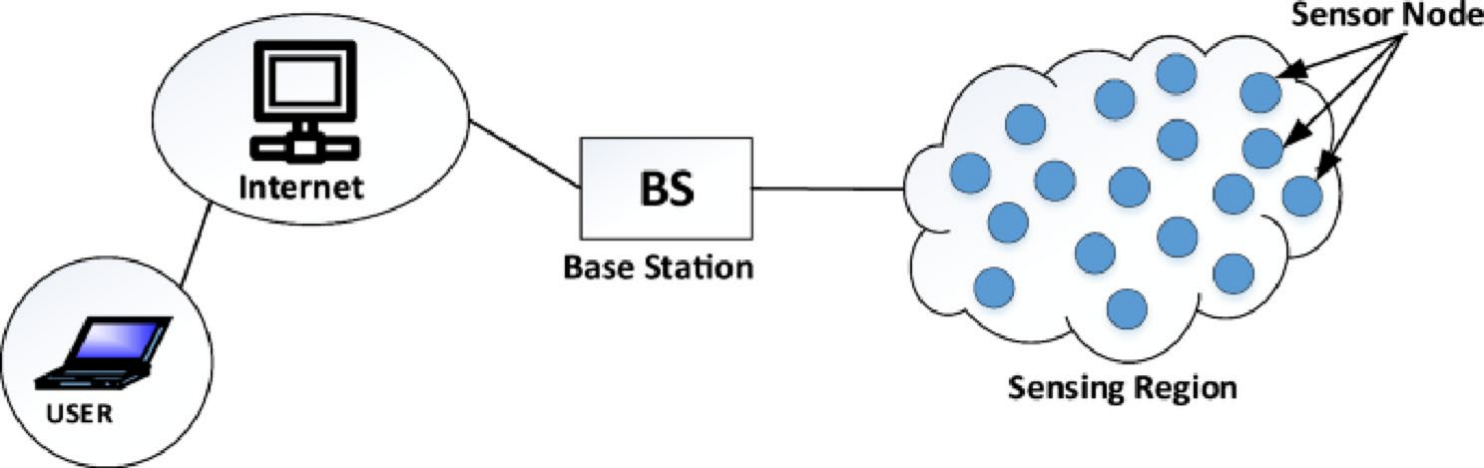
Wireless sensor networks^15^.

A Wireless Sensor Network (WSN)^1–3^ consist of large numbers of spatially distributed sensing nodes that cooperatively monitor environmental or physical conditions such as temperature, humidity, pressure, motion, vibration, or pollution levels. These nodes are typically equipped with sensing, processing, and short-range wireless communication capabilities, enabling them to transmit the collected data either directly or through multiple hops to a centralized Base Station (BS) for further processing (Fig. 1).

WSNs are increasingly used in diverse applications, including environmental monitoring, precision agriculture, disaster management, industrial automation, healthcare monitoring, smart cities, and Internet of Things (IoT) infrastructures. Their flexibility and wide deployment potential have made them a core enabling technology in modern intelligent systems.

However﻿﻿﻿, ﻿﻿one of the most critical challenges in WSNs is energy consumption, as most sensor nodes operate on non-rechargeable batteries and are often deployed in inaccessible or dangerous environments. This makes energy efficiency the most essential design goal in WSN communication protocols, especially routing protocols, which are responsible for determining how sensed data is forwarded from nodes to the BS. Efficient routing strategies must therefore focus on reducing communication cost, balancing energy consumption, and prolonging the network lifetime, while adapting to dynamic topology changes, node failures, and uneven traffic distribution.

Depending on the network architecture, WSNs routing protocols can be classified into flat-based, hierarchical (cluster-based), and location-based approaches^16^. In flat routing, all nodes are treated equally, whereas hierarchical routing organizes nodes into clusters to reduce redundant communication and conserve energy. Location-based protocols leverage the geographical positions of nodes to optimize data forwarding.

### Low-energy adaptive clustering hierarchy routing protocol and its variants

**Fig. 2:**
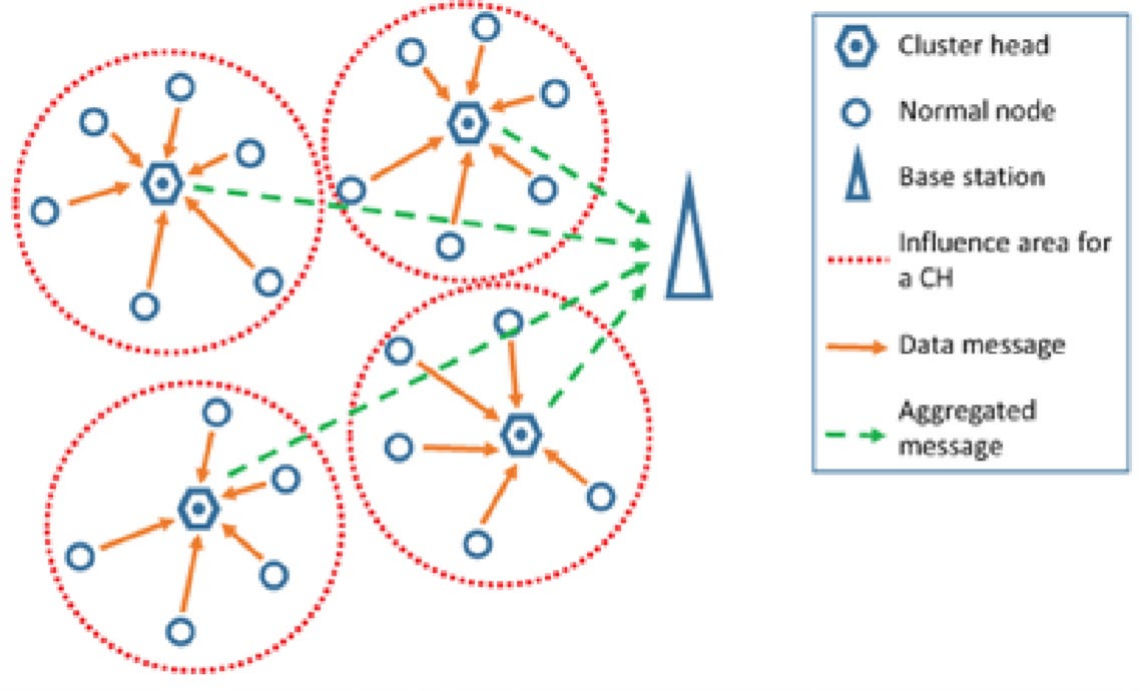
Leach clustering operation^8^.

The Low-Energy Adaptive Clustering Hierarchy (LEACH) protocol^6–8^ is the pioneering hierarchical routing protocol developed for Wireless Sensor Networks (WSNs), with the objective of optimizing energy consumption and extending the overall network lifetime. LEACH organizes sensor nodes into clusters, where each cluster is managed by a Cluster Head (CH) responsible for aggregating and forwarding data to the Base Station (BS). It employs a fully distributed clustering mechanism that operates in rounds. During each round, nodes probabilistically elect themselves as CHs. The elected CHs then collect data from nearby member nodes, perform data aggregation, and subsequently forward the aggregated information to the BS (Fig. 2). Every round is divided into two phases: a setup phase, during which clusters are formed and CHs are elected, and a steady-state phase, during which data transmission takes place. To ensure balanced energy consumption and mitigate premature node depletion, LEACH periodically rotates the CH role among the nodes. However, the CH selection process is entirely randomized, without consideration of parameters such as residual energy or node location, which may result in suboptimal energy utilization.

Consequently, numerous enhancements have been proposed to improve the performance of the conventional LEACH protocol. The following subsections describe some of the centralized variants derived from the LEACH framework.

#### Conventional LEACH-based enhancements

These approaches enhance the standard LEACH protocol through modifications in cluster-head selection logic, load balancing, sleep scheduling, or multi-hop forwarding. They primarily aim to optimize energy consumption while maintaining conventional rule-based decision mechanisms, without incorporating artificial intelligence or learning-based adaptation. The following are some of these approaches^9,17–28^:

 Centralized LEACH (LEACH-C)^9^ is an improved version of the LEACH routing protocol. Unlike the original LEACH, which allows sensor nodes to autonomously form clusters, LEACH-C assigns control to the BS. During the setup phase, all nodes transmit their energy level and location (if mobile) to the BS, which then selects the CHs and informs all nodes. In the steady-state phase, the nodes send their data to the CHs. Each CH aggregates the collected data and forwards it to the BS. LEACH-C employs a TDMA schedule to avoid data collisions. It provides improved energy savings, balanced workload distribution, and extended network lifetime. However, it is dependent on the BS and may not perform efficiently in very large or highly dynamic networks. Furthermore, each node must be aware of its location, which may require additional hardware. LEACH-C is best suited for static networks where energy efficiency and long-term operation are critical, such as in agricultural or environmental monitoring applications.

 Fuzzy logic-based centralized LEACH (Fuzzy LEACH-C)^17^ employs fuzzy logic to enhance CH selection. It evaluates multiple parameters, including residual energy, distance to the BS, and node centrality within the cluster, using fuzzy inference rules. This intelligent decision-making mechanism enables the BS to select CHs more adaptively and accurately, leading to more balanced cluster formation and reduced energy consumption. The integration of fuzzy logic reduces randomness in CH selection and improves overall network performance, particularly in heterogeneous and dynamic environments. However, this approach increases computational complexity at the BS, requiring additional processing time and resources.

 Distributed energy-efficient clustering (DEEC)^18^, is designed for heterogeneous wireless sensor networks. DEEC preserves LEACH’s distributed clustering architecture, but replaces CHs’ random selection with an energy-aware decision model. In DEEC, each node estimates its probability of becoming a cluster head based on its residual energy and the average network energy at each round. This probabilistic approach ensures that nodes with higher remaining energy are more likely to become cluster heads, thereby balancing energy consumption across the network and extending overall network lifetime. Consequently, it improves stability and efficiency in heterogeneous environments.

 Balanced LEACH (LEACH-B)^19^ focuses on achieving more balanced energy usage among sensor nodes by refining the CH selection process. Instead of considering only residual energy, LEACH-B also incorporates the distance between nodes and the BS. This dual consideration helps prevent the overuse of nodes that are either too far or have low energy. Its main advantage is the prevention of early energy depletion in specific nodes, resulting in a more balanced network. However, this comes at the cost of increased initial computation, as the BS must evaluate both energy and distance metrics for every node before forming clusters.

 Fixed clustering LEACH (LEACH-F)^20^ simplifies the clustering process by forming fixed clusters at the beginning of the network’s operation. CHs are selected only once during initialization and remain unchanged throughout the network’s lifetime. This significantly reduces setup overhead, thereby saving the energy that would otherwise be consumed in repeated CH selection. LEACH-F benefits from lower energy consumption and a simplified protocol structure. However, it lacks adaptability, as it cannot respond to changes such as node death or mobility, which may lead to performance degradation and loss of communication over time.

 LEACH with sleep scheduling (LEACH-CS)^21^ introduces energy-saving mechanisms by allowing non-CH nodes to enter sleep mode during inactive periods. After cluster formation, the BS assigns time slots not only for data transmission (as in traditional TDMA scheduling) but also for sleep intervals. Nodes that are not scheduled to transmit or receive can power off their radios to conserve energy. This reduces idle listening and prevents unnecessary energy waste. As a result, sleep scheduling helps extend network lifetime, particularly in applications with periodic data reporting and predictable communication patterns. The main drawback of this method is increased latency, as nodes may be asleep when critical events occur.

 LEACH with gateway nodes (LEACH-G)^22^ is designed for large-scale wireless sensor networks. It introduces an intermediate layer of gateway nodes between the CHs and the BS. After aggregating data from their cluster members, CHs transmit this data to a gateway node, which then forwards it to the BS. This approach reduces the direct communication range required for CHs, thereby conserving energy. The key advantage of LEACH-G is reduced energy consumption across the network, particularly for distant CHs. However, the inclusion of gateway nodes adds additional complexity and overhead in terms of coordination and route maintenance.

 Split and merge LEACH (LEACH-SM)^23^ introduces a dynamic clustering mechanism in which the BS monitors cluster sizes over time. If a cluster becomes excessively large, it is split into smaller clusters to reduce the communication burden on the CH. Conversely, if a cluster becomes too small due to node failures or energy depletion, it is merged with a neighboring cluster to maintain connectivity. This adaptive strategy helps maintain efficient energy utilization and balanced communication load, thereby extending network lifetime. The main challenges are the additional computational burden on the BS and the potential delay caused by frequent re-clustering through the split-and-merge mechanism.

 Energy-efficient cluster head selection with adaptive threshold^24^ introduces a dynamic CH selection threshold that adapts based on each node’s residual energy rather than using a fixed probability. This reduces the likelihood of low-energy nodes becoming CHs and ensures a more balanced rotation of CH roles, resulting in enhanced network stability and prolonged lifetime.

 Energy and load-balanced LEACH (EL-LEACH)^25^ incorporates both residual energy and node load into the CH selection process. CH selection considers not only the residual energy of nodes but also the communication load they handle. It further prevents overloading specific nodes by evenly distributing communication and aggregation tasks. This approach helps prevent early energy depletion in heavily loaded nodes, resulting in improved energy balance, a longer stability period, and ultimately an extended overall network lifetime. However, the inclusion of load awareness increases the computational complexity during the cluster formation stage.

 LEACH-C with unequal clustering^26^ is designed to address energy imbalance, particularly in networks where the BS is located far from many nodes. In this approach, the BS forms clusters of unequal sizes based on the proximity of CHs to itself. Nodes closer to the BS are assigned to smaller clusters to reduce their workload, as they are more likely to forward additional traffic from distant clusters. Conversely, nodes located farther from the BS are grouped into larger clusters to balance overall energy consumption. This unequal clustering strategy prevents nodes near the BS from depleting their energy too quickly, thereby extending network lifetime and improving scalability. However, its main limitation is the added complexity resulting from the use of multiple metrics and the dual CH selection process.

 Distance-distributed energy-efficient clustering (D-DEEC)^27^ protocol is an improvement over DEEC, retaining its distributed and energy-adaptive structure while incorporating distance awareness into the cluster-head election process. In D-DEEC, each node considers both its residual energy and its distance to the base station (BS) or the average network distance when determining its cluster-head probability. This modification minimizes the excessive energy depletion of nodes located far from the BS, mitigating the “energy hole” problem common in large-scale networks. Additionally, D-DEEC introduces a sleep/awake mechanism and a threshold energy model to further reduce redundant transmissions and balance energy usage.

 Modified RCH-LEACH (MRCH)^28^ introduces an enhanced CH reselection mechanism that considers both residual energy and a Relative Communication Hierarchy (RCH) factor. This approach more evenly distributes the communication load among nodes, prioritizing those with higher stability and optimal positioning. As a result, MRCH reduces premature node death; particularly for nodes located near the BS; and extends network lifetime.

#### Machine-learning-enhanced variants of LEACH

While conventional LEACH variants rely on fixed heuristics, recent research has shifted toward integrating intelligent learning mechanisms to enhance adaptability under dynamic or heterogeneous network conditions. The following protocols incorporate machine-learning or predictive optimization to enable more context-aware and efficient cluster-head selection. Recent studies have introduced machine-learning into LEACH-based routing to further optimize clustering and energy consumption. The following are some of these approaches^10,13,29–33^:

 Threshold-sensitive energy efficient network enhanced with K-Nearest Neighbor (TEEN-KNN)^29^ integrates a K-Nearest Neighbor (K-NN) learning mechanism with a reactive threshold-based data forwarding strategy. Instead of continuous data broadcasting, it dynamically evaluates sensed values against hard and soft threshold levels while using K-NN, to intelligently predict the most suitable node to act as a CH. This allows the protocol to adapt CH selection not only based on current residual energy and node proximity but also on historical behavior patterns and traffic relevance, making TEEN-KNN highly efficient in event-driven and rapidly changing WSN environments.

 Reinforcement learning-based LEACH (LEACH-RL)^30^ employs a Q-learning mechanism to dynamically select cluster heads based on long-term expected rewards rather than single-round heuristics. The reward function incorporates key parameters such as residual energy, distance to the base station, and local node density, allowing the algorithm to gradually learn CH-selection policies that maximize network lifetime. Although this RL-based method significantly reduces premature CH depletion, it requires continuous state monitoring, Q-table updates, and additional control exchanges, which introduce computational and communication overhead.

Neural network improved LEACH (NN-ILEACH)^31^ uses a supervised neural network to score nodes for CH candidacy based on energy and topology features, and couples this with an Energy Hole Removing Mechanism (EHORM) module that mitigates energy holes by adaptively balancing CH placement and power. The approach replaces purely random CH rotation with data-driven selection, yielding substantial increases in network lifetime, throughput and packet delivery ratio. The major trade-offs are the need for offline training (or periodic retraining) and extra computation/control logic to run the trained model and the EHORM, which may limit applicability on extremely constrained nodes unless executed at the BS.

 Intra cluster gateway node particle swarm optimization-genetic algorithm (ICGW-PSOGA)^32^ is an energy-efficient solution that addresses energy wastage caused by residual nodes and long communication distances, by determining the optimal placement of Intra-Cluster Gateway (IC-GW) nodes and the BS. This optimal placement is achieved through a hybrid optimization algorithm that combines Particle Swarm Optimization (PSO) and Genetic Algorithm (GA) to minimize both intra-cluster and inter-cluster communication overhead, thereby extending the overall network lifetime.

 K-means-based LEACH^10^, integrates K-means clustering into the LEACH-C protocol^9^ to enhance cluster formation by grouping sensor nodes based on their spatial proximity. In this approach, the BS employs K-means to generate compact and balanced clusters, thereby minimizing intra-cluster communication distances and improving overall energy efficiency. K-means is selected due to its lightweight and deterministic nature, its lack of training requirements, and its seamless compatibility with the centralized scheduling mechanism of LEACH-C, making it particularly suitable for energy-constrained WSN environments. The combination of LEACH-C with K-means—commonly referred to as K-Means-Based LEACH; has demonstrated superior performance, achieving a longer network lifetime and a higher number of successfully transmitted packets compared to Mean-Shift and closeness-centrality-based clustering mechanisms.

 DEEC with K-Means clustering (DEEC-KM)^13^ is an enhanced variant of DEEC that improves cluster formation in heterogeneous WSNs by integrating centralized K-means clustering at the BS. At the beginning of each round, the BS groups alive nodes into spatially balanced clusters, while CH selection and data transmission remain distributed; CH selection is performed distributively, where the node with the highest residual energy becomes the cluster head. The number of clusters adapts to the number of surviving nodes, improving energy distribution and reducing communication distances. Although DEEC-KM enhances lifetime and packet delivery, it introduces additional BS computation and requires nodes to know their positions, increasing protocol complexity compared to standard DEEC.

 Deep learning-based hybrid energy-efficient distributed clustering (DL-HEED)^33^ employs a deep learning–driven decision model that analyzes multidimensional features such as residual energy, node centrality, distance to the BS, and historical communication load to enable more informed and globally optimized CH selection. Unlike conventional HEED, which relies solely on residual energy and local information, DL-HEED continuously learns and adapts to evolving network dynamics, resulting in more accurate CH prediction, an extended stability period, and superior performance under heterogeneous or high-traffic WSN conditions.

#### Hybrid LEACH variants

Hybrid LEACH variants integrate multiple optimization techniques; such as clustering, multi-hop routing, or mobile BS assistance; within a unified framework. Instead of relying on a single strategy, they combine complementary mechanisms to improve scalability, reduce transmission cost, and balance network load. The following are some of these approaches^34,35^:

 Energy-efficient hybrid clustering and hierarchical routing (EEHCHR)^34^ combines intelligent machine-learning–inspired clustering with hierarchical multi-hop forwarding. EEHCHR jointly considers residual energy, inter-node distance, and expected forwarding load to dynamically assign CHs and optimize multi-level data aggregation. This hybrid strategy effectively balances the data forwarding load, prevents early depletion of central relay nodes, and significantly enhances scalability and network lifetime in dense or large-scale WSN deployments.

 Hybrid rendezvous clustering model^35^ provides an energy-efficient solution for data collection in multi-sink wireless sensor networks by integrating traditional clustering with sink (BS) mobility and rendezvous scheduling. Rather than requiring every CH to transmit directly to a fixed sink, the protocol determines optimal rendezvous points where mobile sinks temporarily stop to collect aggregated data. This approach reduces long-distance transmissions, balances communication load, and substantially improves scalability in large-area deployments. However, the protocol depends on precomputed sink trajectories (travel path of a mobile base station to collect data efficiently from sensor nodes) and rendezvous timing, which may constrain adaptability in rapidly changing network environments.

### Other energy-efficient non-LEACH routing protocols

In addition to LEACH-based methods, several routing protocols enhance energy efficiency through alternative strategies, including mobile sink (BS) guidance, optimal gateway placement, delay awareness, or cognitive spectrum sensing. These approaches provide energy-saving solutions that extend beyond traditional cluster-head rotation mechanisms. The following are some of these approaches^36–38^:

 Energy efficient routing in wireless sensor network based mobile sink guided by stochastic hill climbing^36^ enables energy-efficient routing by combining geographic data relaying with an adaptively BS. Rather than following a predetermined trajectory, the BS’s movement is optimized using a stochastic hill-climbing search to reduce hop count and energy consumption during data forwarding. The BS position is dynamically updated based on nodes’ sensing and transmission rates, helping to minimize relay overhead and prolong network lifetime. However, this method may become trapped in local optima due to the inherent characteristics of hill-climbing optimization.

 Score-based link delay-aware routing (SBLDAR)^37^ is a delay-sensitive routing protocol that determines forwarding paths using a link score that incorporates expected transmission delay, link quality, and residual energy. Instead of relying on clustering, SBLDAR dynamically evaluates real-time link conditions to prioritize routes that minimize end-to-end latency, making it well-suited for mission-critical applications such as healthcare and industrial monitoring where timely data delivery is crucial. Although it enhances path stability and reduces delay under varying network conditions, its lack of clustering and data aggregation can cause frequently selected nodes to deplete their energy quickly.

 Cognitive radio-based energy optimization approach^38^ improves WSN performance by enabling nodes to dynamically sense available spectrum and intelligently select both the optimal frequency band and the most energy-efficient forwarding route. Instead of relying on fixed channels, nodes opportunistically transmit using available spectrum to avoid interference and reduce retransmissions. By jointly optimizing spectrum selection and next-hop routing, the protocol significantly reduces overall communication energy. However; the additional sensing and decision-making overhead increases processing complexity and may not be suitable for ultra-low-power sensor devices.

A comparison of the above protocols is presented in Table 1.

**Table 1:** Comparison of LEACH-based and other energy-efficient routing protocols in WSNs.

Protocol	Technique	Energy strategy	Key advantage	Limitation
LEACH^6–8^	Probabilistic clustering	CH rotation	Simple and fully distributed design	Random CH selection may lead to energy imbalance
LEACH-C^9^	Centralized clustering	BS-assisted optimal CH selection	More balanced clusters and higher efficiency	Requires node location; limited scalability
Fuzzy LEACH-C^17^	Fuzzy logic inference	Multi-metric CH selection	Adaptive and intelligent CH decisions	Increased computational load at BS
DEEC^18^	Energy-proportional CH selection	Residual energy of each node	Balances energy consumption among nodes & extends network lifetime	Ignores node distance to BS, which may cause higher energy use for distant CHs
LEACH-B^19^	Distance + energy weighting	Balanced CH load	Prevents early node depletion	Higher setup overhead
LEACH-F^20^	Fixed clustering	No re-clustering overhead	Very low setup cost	Lacks adaptability to node failures
LEACH-CS^21^	Sleep scheduling	Radio sleep intervals	Conserves energy during idle periods	May introduce latency
LEACH-G^22^	Gateway layer	Multi-hop via gateways	Scales efficiently for large networks	Adds routing complexity
LEACH-SM^23^	Split and merge mechanism	Adaptive cluster resizing	Maintains energy balance over time	Frequent re-clustering increases overhead
Adaptive threshold LEACH^24^	Dynamic CH threshold based on residual energy	Energy-aware CH probability	Prevents low-energy nodes from becoming CH	Lacks predictive or adaptive intelligence
EL-LEACH^25^	Load-aware balancing	Even traffic distribution	Prevents CH overload	Higher computational complexity
LEACH-C (unequal clustering)^26^	Distance-aware cluster sizing	Unequal cluster sizes	Mitigates energy hotspots near BS; balances cluster energy	More complex cluster formation
D-DEEC^27^	Energy- & distance-aware cluster-head selection	Residual energy & distance to the BS, with a sleep/awake mechanism	Reduces energy hole near base station and further extends network lifetime	Adds computation overhead and depends on accurate distance estimation
MRCH-LEACH^28^	RCH + residual energy	Load-balanced CH rotation	Reduces premature node failure near BS	Heuristic-only; no learning capability
TEEN-KNN^29^	K-NN learning + threshold	Adaptive sensing thresholds	Highly efficient for event-driven networks; reduces false alarms	Sensitive to threshold tuning, High computation
LEACH-RL^30^	Reinforcement Learning (Q-learning-based CH selection)	Reward functions based on residual energy, distance & node density	Adapts CH selection dynamically and avoids premature CH death, improving lifetime	Continuous state monitoring & Q-table updates; computational & communication overhead
NN-ILEACH^31^	Supervised Neural Network + EHORM	CH scoring using learned model (residual energy); EHORM balances energy holes	Strong lifetime and packet-delivery improvements; adaptive CH selection	Requires training & model execution; extra computation/control overhead
ICGW-PSOGA^32^	Hybrid PSO-GA optimal gateway placement	Minimizes intra- and inter-cluster transmissions	Reduces CH overload and long-distance transmissions	Requires centralized topology information; metaheuristic complexity
K-means LEACH^10^	K-means clustering	Distance-minimized clusters	Very compact & energy-efficient clusters	Centralized computation; limited scalability
DEEC-KM^13^	K-means clustering + residual-energy-based CH	K-means for cluster formation; residual energy for CH selection	Spatially balanced clusters; improves energy distribution, network lifetime	Computational overhead at BS; nodes must know their positions
DL-HEED^33^	Deep learning CH selection	Intelligent energy modeling	Optimized CH selection accuracy; extends network lifetime	Requires training; higher processing overhead
EEHCHR^34^	Hierarchical + adaptive ML	Combined clustering logic	Strong scalability; improved network lifetime	More complex system integration; requires stable topology
Hybrid rendezvous clustering^35^	Clustering + mobile sink rendezvous	Reduces long-distance CH-to-sink transmissions	Highly scalable; efficient for multi-sink WSNs	Requires preplanned sink movement; high latency
Mobile sink + stochastic hill climbing^36^	Geographic routing + adaptive BS relocation	Reduces hop count through optimized sink movement	Minimizes relay load; extends network lifetime	Risk of local optima; depends on mobility control
SBLDAR^37^	Link score-based QoS routing	Selects low-delay, reliable links	Suitable for real-time and delay-sensitive applications; ensures link reliability	No clustering; may lead to higher overall energy consumption
Cognitive radio energy optimization^38^	Dynamic spectrum-aware routing	Selects lowest-energy free spectrum and next-hop route	Avoids collisions and energy waste; highly adaptive	Additional sensing and routing overhead; increased complexity

## Proposed protocol

This section first provides a concise overview of the K-means algorithm, followed by a detailed presentation of the proposed Energy-Driven K-Means-based LEACH Routing Protocol for enhanced lifetime in Wireless Sensor Networks, including its underlying rationale and implementation methodology.

### K-means based LEACH routing protocol

The K-Means-Based Leach routing protocol^10^, employs the traditional K-means clustering algorithm within the centralized LEACH-C framework. K-means is a deterministic, unsupervised machine-learning algorithm; it requires no training data, it converges quickly; and has a complexity of O(n·k·i), where n is the number of data points in the dataset, k is the number of clusters and i is the number of iterations the algorithm takes to converge.

K-means clustering^12,13^ can be formulated as the following optimization problem:

- Given.
  - $\:S=\left\{{s}_{1},{s}_{2},\:\dots\:\dots\:\:{s}_{n}\right\}$: Set of $\:n$ data points in a two-dimensional space representing the sensor nodes’ location.
  - $\:K$: Number of clusters.
  - $\:{c}_{j}$: Centroid of cluster $\:j$, i.e. the Cluster Head (CH) of cluster $\:j$.
  - $\:{C}_{j}$: Set of points (sensor nodes) assigned to cluster$\:\:j$.
- Find K cluster centers $\:C=\{{c}_{1},\:...,\:{c}_{K}\}$ and an assignment function $\:f:\:S\to\:C$ that minimize the total squared Euclidean distance between each sensor node and its assigned CH.
- Objective function:

1 $$\:{\boldsymbol{m}\boldsymbol{i}\boldsymbol{n}}_{\left\{{\boldsymbol{C}}_{\boldsymbol{j}}\right\},\left\{{\boldsymbol{c}}_{\boldsymbol{j}}\right\}}\sum\:_{\boldsymbol{j}=1}^{\boldsymbol{K}}\sum\:_{{\boldsymbol{s}}_{\boldsymbol{i}}\in\:{\boldsymbol{C}}_{\boldsymbol{j}}}{\left\|{\boldsymbol{s}}_{\boldsymbol{i}}-{\boldsymbol{c}}_{\boldsymbol{j}}\right\|}^{2}$$

where: $\:{s}_{i}$ is sensor node $\:i$ location.

$$\:{C}_{i}\cap\:{C}_{j}=\varnothing\:,\:\:\forall\:\:i\ne\:j\:\bigcup\:_{j=1}^{K}{C}_{j}=S$$

$$\:{c}_{j}=\raisebox{1ex}{$1$}\!\left/\:\!\raisebox{-1ex}{$\left|{C}_{j}\right|$}\right.\sum\:_{{s}_{i}\in\:{C}_{j}}{s}_{i}$$

$\:{c}_{j}$ is the mean of the sensor nodes in cluster $\:{C}_{j}$.

Although the classical K-means algorithm relies on Euclidean distance, numerous studies have explored alternative distance metrics to adapt the algorithm to various application domains. For example:

- K-Medians clustering^39^ replaces the Euclidean distance with Manhattan distance by using medians instead of means as cluster centers, offering improved robustness to outliers.
- Spherical K-means^40^ employs cosine similarity instead of Euclidean distance to better accommodate high-dimensional sparse data.
- Minkowski distance-based K-means^41^ employs the Minkowski distance, a generalized metric that encompasses multiple distance norms; allowing K-means to flexibly adapt to datasets with different similarity requirements.

### Proposed energy-driven K-means-based LEACH routing protocol

**Fig. 3:**
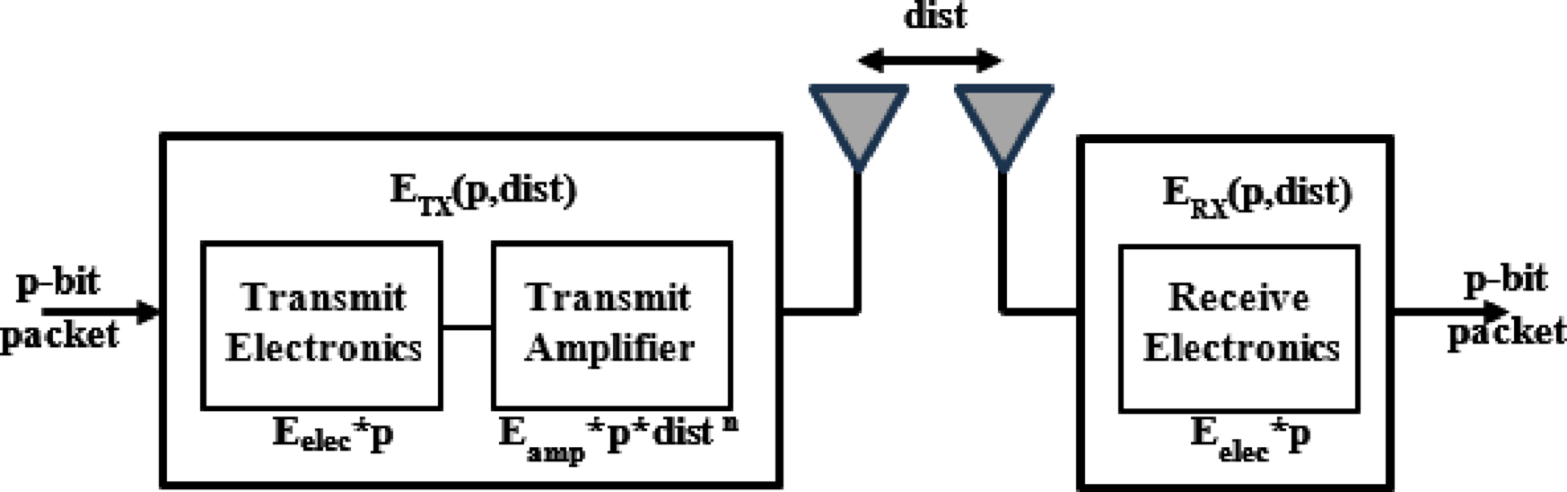
Radio energy dissipation model.

Minimizing Euclidean distance alone does not necessarily guarantee minimal actual transmission energy. In WSNs, communication energy increases nonlinearly with distance; particularly beyond the free-space threshold; making energy consumption a more appropriate optimization objective than geometric distance. In this context, clustering is not solely based on spatial proximity; the primary goal is to minimize energy expenditure during communication between sensor nodes and their respective Cluster Heads (CHs). The communication energy used in this study is governed by the Radio Energy Dissipation Model^9,14^ (Fig. 3).

- Transmitter energy consumption

In the radio energy dissipation model, the transmitter expends energy for both the radio electronics and the power amplifier, with the transmission power adapted according to the distance to the receiving node. Consequently, the transmitter energy consumption is calculated by considering these two components:

- Electronic energy ($\:{{E}}_{{e}{l}{e}{c}}$): Energy consumed per bit for operations such as digital coding, modulation, filtering, and signal spreading.
- Amplification energy ($\:{{E}}_{{a}{m}{p}}$): Energy consumed per bit for power amplification, dependent on the transmission distance and the acceptable bit-error rate.

A distance-based path loss model is applied, using different exponents depending on the distance $\:d$ with a threshold ($\:{d}_{O}$).

- If $\:\boldsymbol{d}<{\boldsymbol{d}}_{\boldsymbol{o}}$, a free-space model with a path loss exponent of 2 is used.
- If $\:\boldsymbol{d}\ge\:{\boldsymbol{d}}_{\boldsymbol{o}}$, a multipath fading model with an exponent of 4 is used.

So, the energy required to transmit a $\:p$-bit packet over a distance $\:d$ is:

2 $$\:{\boldsymbol{E}}_{\boldsymbol{T}\boldsymbol{x}}\left(\boldsymbol{p},\boldsymbol{d}\right)=\left\{\begin{array}{cc}\boldsymbol{p}\boldsymbol{*}{\boldsymbol{E}}_{\boldsymbol{e}\boldsymbol{l}\boldsymbol{e}\boldsymbol{c}}+\boldsymbol{p}\boldsymbol{*}{\boldsymbol{E}}_{\boldsymbol{f}\boldsymbol{s}}\boldsymbol{*}{\boldsymbol{d}}^{2},\:\:\:\:\:\:&\:\boldsymbol{d}<{\boldsymbol{d}}_{\boldsymbol{o}}\:(\boldsymbol{f}\boldsymbol{r}\boldsymbol{e}\boldsymbol{e}-\boldsymbol{s}\boldsymbol{p}\boldsymbol{a}\boldsymbol{c}\boldsymbol{e}\:\boldsymbol{m}\boldsymbol{o}\boldsymbol{d}\boldsymbol{e}\boldsymbol{l})\\\:\boldsymbol{p}\boldsymbol{*}{\boldsymbol{E}}_{\boldsymbol{e}\boldsymbol{l}\boldsymbol{e}\boldsymbol{c}}+\boldsymbol{p}\boldsymbol{*}{\boldsymbol{E}}_{\boldsymbol{a}\boldsymbol{m}\boldsymbol{p}}\boldsymbol{*}{\boldsymbol{d}}^{4},\:\:\:&\:\boldsymbol{d}\ge\:{\boldsymbol{d}}_{\boldsymbol{o}}\:(\boldsymbol{m}\boldsymbol{u}\boldsymbol{l}\boldsymbol{t}\boldsymbol{i}-\boldsymbol{p}\boldsymbol{a}\boldsymbol{t}\boldsymbol{h}\:\boldsymbol{m}\boldsymbol{o}\boldsymbol{d}\boldsymbol{e}\boldsymbol{l})\end{array}\right.\:$$

where: $\:{E}_{Tx}\left(p,d\right)\:$is the energy consumed to send a $\:p$-bit packet across a distance $\:d$.

$\:{E}_{elec}$ is the electronic energy per bit.

$\:{E}_{fs}$ and $\:{E}_{amp}\:$are the amplifier parameters for free-space and multipath models, respectively.

$\:{d}_{o}\:$is the threshold distance that determines when to switch between models. It is calculated as follows:

3 $$\:{\boldsymbol{d}}_{\boldsymbol{O}}=\sqrt{\frac{{\boldsymbol{E}}_{\boldsymbol{f}\boldsymbol{s}}}{{\boldsymbol{E}}_{\boldsymbol{a}\boldsymbol{m}\boldsymbol{p}}}}$$

b. Receiver energy consumption

The receiver consumes energy only for radio electronics, as no amplification is required. Therefore, the energy needed to receive a p-bit packet is:

4 $$\:{\boldsymbol{E}}_{\boldsymbol{R}\boldsymbol{x}}\left(\boldsymbol{p},\boldsymbol{d}\right)=\boldsymbol{p}\boldsymbol{*}{\boldsymbol{E}}_{\boldsymbol{e}\boldsymbol{l}\boldsymbol{e}\boldsymbol{c}}$$

where: $\:{E}_{Rx}\left(p,d\right)\:$is the energy consumed to receive a $\:p$-bit packet across a distance $\:d$.

Reception energy is generally lower than transmission energy due to the absence of power amplification.

c. Total communication energy

The total communication energy between two nodes is the sum of transmission and reception energy:

$$\:{\boldsymbol{E}}_{\boldsymbol{C}\boldsymbol{o}\boldsymbol{m}}\left(\boldsymbol{p},\boldsymbol{d}\right)={\boldsymbol{E}}_{\boldsymbol{T}\boldsymbol{x}}\left(\boldsymbol{p},\boldsymbol{d}\right)+{\boldsymbol{E}}_{\boldsymbol{R}\boldsymbol{x}}\left(\boldsymbol{p},\boldsymbol{d}\right)$$

$$\:{\boldsymbol{E}}_{\boldsymbol{C}\boldsymbol{o}\boldsymbol{m}}\left(\boldsymbol{p},\boldsymbol{d}\right)=\left\{\begin{array}{cc}2\boldsymbol{p}\boldsymbol{*}{\boldsymbol{E}}_{\boldsymbol{e}\boldsymbol{l}\boldsymbol{e}\boldsymbol{c}}+\boldsymbol{p}\boldsymbol{*}{\boldsymbol{E}}_{\boldsymbol{f}\boldsymbol{s}}\boldsymbol{*}{\boldsymbol{d}}^{2},\:\:\:\:\:\:&\:\boldsymbol{d}<{\boldsymbol{d}}_{\boldsymbol{o}}\:(\boldsymbol{f}\boldsymbol{r}\boldsymbol{e}\boldsymbol{e}-\boldsymbol{s}\boldsymbol{p}\boldsymbol{a}\boldsymbol{c}\boldsymbol{e}\:\boldsymbol{m}\boldsymbol{o}\boldsymbol{d}\boldsymbol{e}\boldsymbol{l})\\\:2\boldsymbol{p}\boldsymbol{*}{\boldsymbol{E}}_{\boldsymbol{e}\boldsymbol{l}\boldsymbol{e}\boldsymbol{c}}+\boldsymbol{p}\boldsymbol{*}{\boldsymbol{E}}_{\boldsymbol{a}\boldsymbol{m}\boldsymbol{p}}\boldsymbol{*}{\boldsymbol{d}}^{4},\:\:\:&\:\boldsymbol{d}\ge\:{\boldsymbol{d}}_{\boldsymbol{o}}\:(\boldsymbol{m}\boldsymbol{u}\boldsymbol{l}\boldsymbol{t}\boldsymbol{i}-\boldsymbol{p}\boldsymbol{a}\boldsymbol{t}\boldsymbol{h}\:\boldsymbol{m}\boldsymbol{o}\boldsymbol{d}\boldsymbol{e}\boldsymbol{l})\end{array}\right.$$

5 $$\:{\boldsymbol{E}}_{\boldsymbol{C}\boldsymbol{o}\boldsymbol{m}}\left(\boldsymbol{p},\boldsymbol{d}\right)=2\boldsymbol{p}\boldsymbol{*}{\boldsymbol{E}}_{\boldsymbol{e}\boldsymbol{l}\boldsymbol{e}\boldsymbol{c}}+\boldsymbol{p}.\left\{\begin{array}{cc}{\boldsymbol{E}}_{\boldsymbol{f}\boldsymbol{s}}\boldsymbol{*}{\boldsymbol{d}}^{2},\:\:\:&\:\boldsymbol{d}<{\boldsymbol{d}}_{\boldsymbol{o}}\:\\\:{\boldsymbol{E}}_{\boldsymbol{a}\boldsymbol{m}\boldsymbol{p}}\boldsymbol{*}{\boldsymbol{d}}^{4},&\:\boldsymbol{d}\ge\:{\boldsymbol{d}}_{\boldsymbol{o}}\end{array}\right.$$

where: $\:{E}_{Com}\left(p,d\right)\:$is the energy consumption for sending and receiving a $\:p$-bit packet across a distance $\:d$.

During each clustering round of LEACH, the parameters *p*, $\:{E}_{elec}$, $\:{E}_{fs}$ and $\:{E}_{amp}\:$are constants shared across all nodes. Therefore; for optimization, the energy expression can be simplified by ignoring constants that do not affect the optimal centroid positions:

6 $$\:{\boldsymbol{E}}_{\boldsymbol{C}\boldsymbol{o}\boldsymbol{m}}\left(\boldsymbol{p},\boldsymbol{d}\right)=\left(\boldsymbol{c}\boldsymbol{o}\boldsymbol{n}\boldsymbol{s}\boldsymbol{t}\boldsymbol{a}\boldsymbol{n}\boldsymbol{t}\right)+\boldsymbol{p}.\left\{\begin{array}{cc}{\boldsymbol{E}}_{\boldsymbol{f}\boldsymbol{s}}\boldsymbol{*}{\boldsymbol{d}}^{2},\:\:\:&\:\boldsymbol{d}<{\boldsymbol{d}}_{\boldsymbol{o}}\:\\\:{\boldsymbol{E}}_{\boldsymbol{a}\boldsymbol{m}\boldsymbol{p}}\boldsymbol{*}{\boldsymbol{d}}^{4},&\:\boldsymbol{d}\ge\:{\boldsymbol{d}}_{\boldsymbol{o}}\end{array}\right.$$

In K-means clustering, the typical objective is to minimize intra-cluster distances:

7 $$\:\boldsymbol{m}\boldsymbol{i}\boldsymbol{n}\sum\:_{\boldsymbol{i}=1}^{\boldsymbol{i}}{\left\|{\boldsymbol{s}}_{\boldsymbol{i}}-{\boldsymbol{c}}_{\boldsymbol{i}}\right\|}^{2}$$

However, in WSNs, the objective is to minimize communication energy rather than geometric distance:

8 $$\:\boldsymbol{min}({\boldsymbol{E}}_{\boldsymbol{C}\boldsymbol{o}\boldsymbol{m}}\left(\boldsymbol{p},\boldsymbol{d}\right))$$

Starting from the transmitter energy expression $\:{E}_{Tx}\left(p,d\right)$ and receiver energy $\:{E}_{Rx}\left(p,d\right)$, and noting that packet size $\:p$ and per-bit electronics energy $\:{E}_{elec}$ are constant across nodes during a round, the per-bit transmission energy is dominated by the amplifier term which scales as $\:{d}^{n}$ (n ∈ {2,4}). Ignoring constant multiplicative factors that do not affect centroid location, we approximate the per-link cost as proportional to $\:{d}^{n}$ for each link and define the energy-proxy as a weighted combination of $\:{d}^{2}$ and $\:{d}^{4}$ terms proportional to the links falling in the free-space and multipath regions respectively.

9 $$\:{\boldsymbol{E}}_{\boldsymbol{P}\boldsymbol{r}\boldsymbol{o}\boldsymbol{x}\boldsymbol{y}}^{\boldsymbol{{\prime\:}}}\left(\boldsymbol{d}\right)=\left\{\begin{array}{cc}{\boldsymbol{d}}^{2},\:&\:\boldsymbol{d}<{\boldsymbol{d}}_{\boldsymbol{o}}\:\\\:{\boldsymbol{d}}^{4},&\:\boldsymbol{d}\ge\:{\boldsymbol{d}}_{\boldsymbol{o}}\end{array}\right.$$

This heuristic penalizes short distances mildly and long distances more heavily, thereby guiding CHs to be positioned closer to nodes to conserve energy.

The main challenge in applying this heuristic within K-means is that the traditional K-Means-Based LEACH algorithm relies on linear Euclidean distances. Direct use of power functions (e.g., d^2^ or d^4^) may distort the distance metric, leading to suboptimal cluster formation. To maintain proportional consistency between the two distance models, one approach is to treat one as the reference (e.g., d) and adjust the other accordingly (e.g. d^2^); or alternatively, use $\:\sqrt{d}$ and d; ensuring uniformity in clustering behavior. The adjusted energy-proxy function is defined as:

10 $$\:{\boldsymbol{E}}_{\boldsymbol{P}\boldsymbol{r}\boldsymbol{o}\boldsymbol{x}\boldsymbol{y}}^{\boldsymbol{{\prime\:}}}\left(\boldsymbol{d}\right)=\left\{\begin{array}{cc}\begin{array}{cc}\sqrt{\boldsymbol{d}},\:&\:\boldsymbol{d}<{\boldsymbol{d}}_{\boldsymbol{o}}\:\\\:\boldsymbol{d},&\:\boldsymbol{d}\ge\:{\boldsymbol{d}}_{\boldsymbol{o}}\end{array}&\:{\:\:\:\boldsymbol{D}}_{\boldsymbol{M}\boldsymbol{u}\boldsymbol{l}\boldsymbol{t}\boldsymbol{i}}\ge\:{\boldsymbol{D}}_{\boldsymbol{f}\boldsymbol{r}\boldsymbol{e}\boldsymbol{e}}\\\:&\:\\\:\begin{array}{cc}\boldsymbol{d},\:&\:\boldsymbol{d}<{\boldsymbol{d}}_{\boldsymbol{o}}\:\\\:{\boldsymbol{d}}^{2},&\:\boldsymbol{d}\ge\:{\boldsymbol{d}}_{\boldsymbol{o}}\end{array}&\:{\:\:\:\boldsymbol{D}}_{\boldsymbol{f}\boldsymbol{r}\boldsymbol{e}\boldsymbol{e}}>{\boldsymbol{D}}_{\boldsymbol{M}\boldsymbol{u}\boldsymbol{l}\boldsymbol{t}\boldsymbol{i}}\end{array}\right.$$

where: $\:{D}_{free}$ and $\:{D}_{Multi}$ represent the number of node-to-node distances that follow the free space-model ($\:\boldsymbol{d}<{\boldsymbol{d}}_{\boldsymbol{o}})$ and the multi-path model ($\:\boldsymbol{d}\ge\:{\boldsymbol{d}}_{\boldsymbol{o}})$, respectively.

Alternatively﻿, the heuristic can be formulated in terms of the proportion of distances that follow the free-space model.

11 $$\:{\boldsymbol{E}}_{\boldsymbol{P}\boldsymbol{r}\boldsymbol{o}\boldsymbol{x}\boldsymbol{y}}^{\boldsymbol{{\prime\:}}}\left(\boldsymbol{d}\right)=\left\{\begin{array}{cc}\begin{array}{cc}\sqrt{\boldsymbol{d}},\:&\:\boldsymbol{d}<{\boldsymbol{d}}_{\boldsymbol{o}}\:\\\:\boldsymbol{d},&\:\boldsymbol{d}\ge\:{\boldsymbol{d}}_{\boldsymbol{o}}\end{array}&\:{{\:\:\:\:\:\boldsymbol{P}\boldsymbol{e}\boldsymbol{r}}_{\boldsymbol{D}}}_{\boldsymbol{f}\boldsymbol{r}\boldsymbol{e}\boldsymbol{e}}\le\:50\boldsymbol{\%}\\\:&\:\\\:\begin{array}{cc}\boldsymbol{d},\:&\:\boldsymbol{d}<{\boldsymbol{d}}_{\boldsymbol{o}}\:\\\:{\boldsymbol{d}}^{2},&\:\boldsymbol{d}\ge\:{\boldsymbol{d}}_{\boldsymbol{o}}\end{array}&\:{{\:\:\:\:\boldsymbol{P}\boldsymbol{e}\boldsymbol{r}}_{\boldsymbol{D}}}_{\boldsymbol{f}\boldsymbol{r}\boldsymbol{e}\boldsymbol{e}}>50\boldsymbol{\%}\end{array}\right.$$

where: $\:{{\boldsymbol{P}\boldsymbol{e}\boldsymbol{r}}_{\boldsymbol{D}}}_{\boldsymbol{f}\boldsymbol{r}\boldsymbol{e}\boldsymbol{e}}$ is the proportion of distances that follow the free-space model ($\:\boldsymbol{d}<{\boldsymbol{d}}_{\boldsymbol{o}})$.

**Fig. 4:**
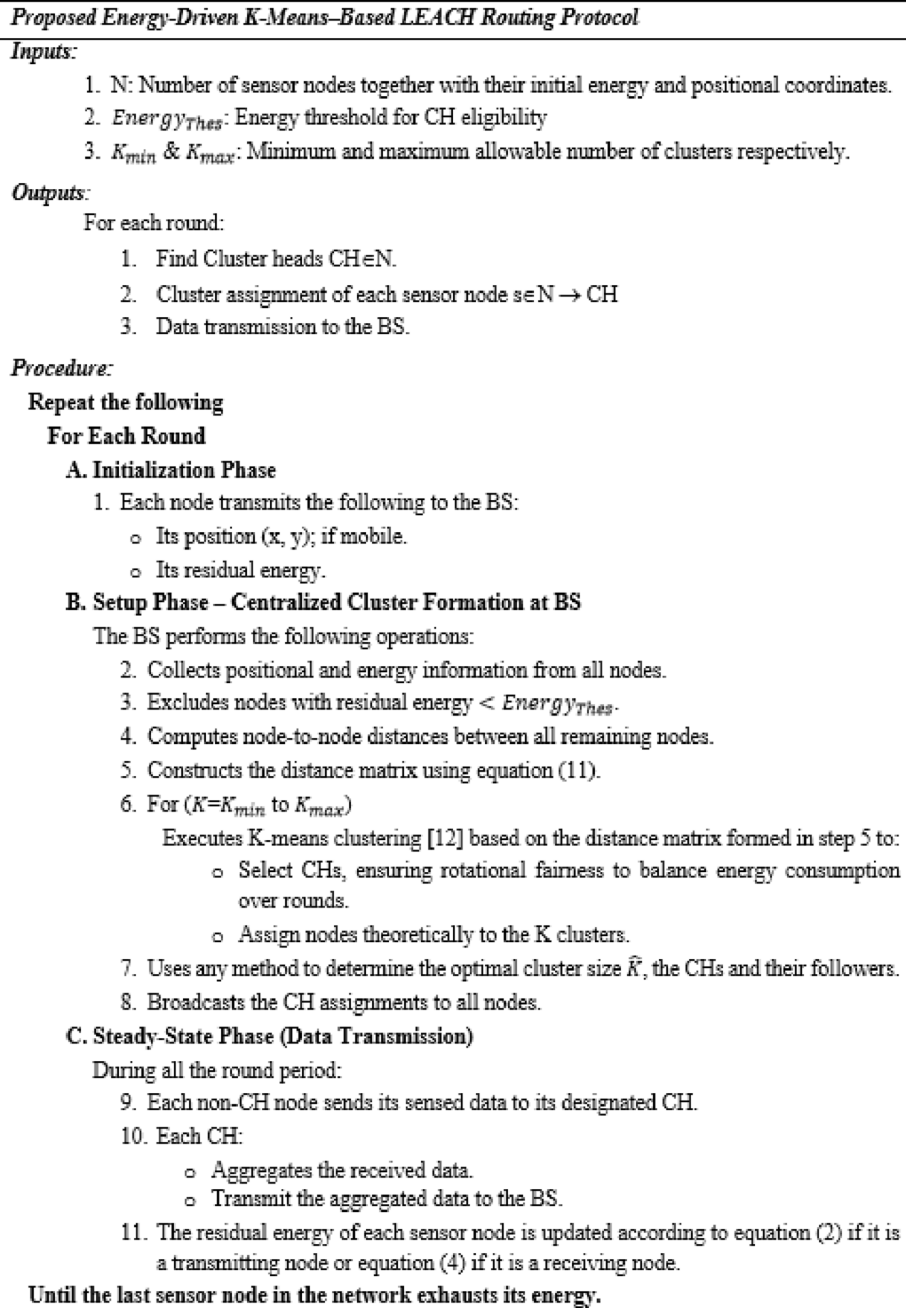
Proposed energy-driven K-means-based LEACH routing protocol.

The proposed Energy-Driven K-Means-based LEACH protocol (Fig. 4) replaces the Euclidean distance with this energy-proxy function during CH selection and node assignment. This Energy-Driven clustering foundation enables the protocol to significantly enhance network lifetime, as demonstrated in Sect. 4.

## Results and analysis

To evaluate the proposed Energy-Driven K-Means-based LEACH routing protocol for Wireless Sensor Networks (WSNs), as described in the preceding section, it was implemented in C + + alongside with the traditional K-Means-based LEACH^10^ and DEEC-KM^13^ routing protocols (The C + + simulation code and the data files used in our experiments are available from the corresponding author upon reasonable request). The evaluation considered multiple deployment areas, and for each area, various network topologies with non-uniformly distributed nodes were tested using different randomly generated packet files. This section is organized into four subsections: the underlying assumptions, the performance metrics used for evaluation, the simulation results along with their analysis, and a brief concluding summary.

### Assumptions

During the development of the simulation program, the following key assumptions were considered:

1. Non-uniform node distribution: The WSN under study is non-uniformly distributed^3^, meaning that sensor nodes are placed irregularly. Such distributions occur due to environmental constraints, for instance, in precision agriculture, where sensors are deployed more densely in drought- or disease-prone areas, while more stable regions require fewer sensors. Application-driven requirements, such as structural health monitoring, may also necessitate denser deployment around critical joints. An example for network topology is illustrated in Fig. 5.

**Fig. 5:**
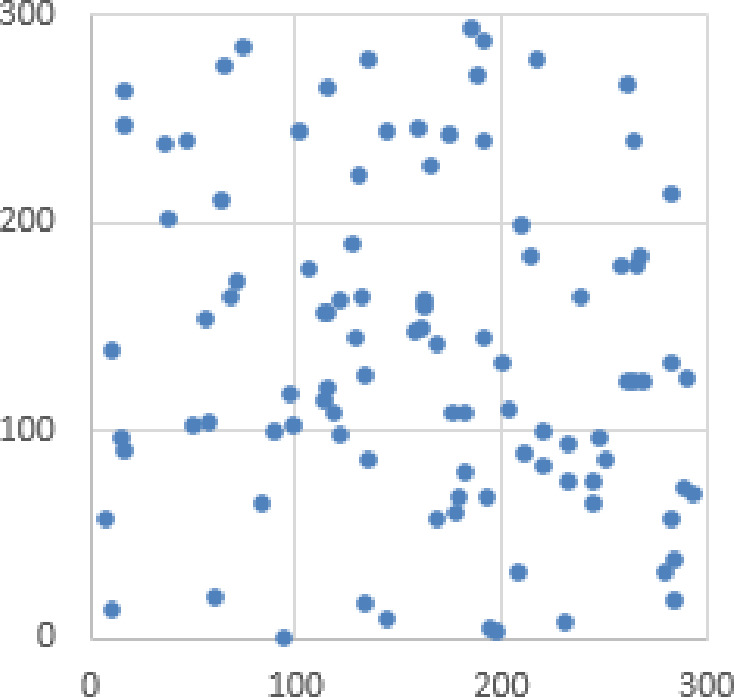
An example of non-uniformly distributed WSN topology.
2. Power control: Transmission power is dynamically adjusted according to the distance between the transmitting and receiving nodes^42^. Greater power is used for longer distances, and lower power for shorter distances. This approach optimizes network performance, reduces energy consumption, extends battery lifetime in WSNs and mobile devices, and ensures sufficient signal strength for reliable communication while minimizing errors.
3. Optimal cluster determination in K-means: The most common methods for determining the optimal number of clusters are the Elbow Method, Silhouette Method, and Gap Statistic^11^. The Elbow Method identifies the point at which adding additional clusters no longer results in a substantial reduction of the Within-Cluster Sum of Squares (WCSS). The Silhouette Method assesses clustering quality by evaluating both the cohesion of points within clusters and the separation between different clusters, with higher Silhouette scores indicating better-defined and well-separated clusters. The Gap Statistic measures clustering performance by comparing the obtained clustering results against those expected from a reference random dataset, providing an indication of the statistical significance of the clustering structure. While any method can be used in the proposed protocol, the Silhouette Method was selected due to its clear interpretability and balance between cluster cohesion (closeness of points within the same cluster) and separation (distance from points in other clusters). The procedure is as follows:
  For each value of cluster $\:K$ number between $\:{K}_{min}$ & $\:{K}_{max}$.
    Execute the K-means clustering process using any of the three compared approaches.
    Compute the silhouette coefficient for each point (sensor node):
      12 $$\:{\boldsymbol{S}\boldsymbol{i}\boldsymbol{l}}_{\boldsymbol{i}}=\frac{{\boldsymbol{b}}_{\boldsymbol{i}}-{\boldsymbol{a}}_{\boldsymbol{i}}}{\boldsymbol{max}({\boldsymbol{a}}_{\boldsymbol{i}},\:{\boldsymbol{b}}_{\boldsymbol{i}})}$$
      where: $\:{Sil}_{i}$ is the silhouette coefficient of node (sensor) $\:{s}_{i}$.
    $\:{a}_{i}$ is the average distance from node (sensor) $\:i$ to all other nodes (sensors) in the *same* cluster (cohesion).
    $\:{b}_{i}$ is the minimum average distance from node (sensor) $\:i$ to all nodes (sensors) in *other* clusters (separation).
  A silhouette coefficient close to 1 indicates a well-clustered node.
  A value near 0 indicates the node lies near a cluster boundary.
  A negative value suggests potential misassignment.
    Compute the average silhouette coefficient for all nodes to evaluate clustering quality for each $\:K$:
      13 $$\:\boldsymbol{S}\boldsymbol{i}\boldsymbol{l}\left(\boldsymbol{K}\right)=\frac{1}{\boldsymbol{n}}\sum\:_{\boldsymbol{i}=1}^{\boldsymbol{n}}{\boldsymbol{S}\boldsymbol{i}\boldsymbol{l}}_{\boldsymbol{i}}$$
      where: $\:Sil\left(K\right)$ is silhouette score using K clusters.
      $\:n\:$is the total number of nodes (sensors) in cluster $\:K$.
  The optimal number of clusters $\:\widehat{K}$ corresponds to the maximum silhouette score $\:Sil\left(K\right)$:
    14 $$\:\widehat{\boldsymbol{K}}=\mathbf{arg}{\boldsymbol{m}\boldsymbol{a}\boldsymbol{x}}_{\boldsymbol{S}\boldsymbol{i}\boldsymbol{l}\left(\boldsymbol{K}\right)}$$
    The silhouette evaluation is performed at the BS at the beginning of each round, so it imposes no additional energy cost on the sensor nodes. Its use is optional; other protocols may rely on alternative methods or even fix the number of clusters based on the number of alive nodes. In our experiments, applying the silhouette method does not influence the relative performance of the compared protocols, as it yields the same clustering outcome when the set of alive nodes remains unchanged.
4. Fair comparison between protocols: To ensure unbiased evaluation between traditional K-Means-Based LEACH and the proposed Energy-Driven K-means-based LEACH protocol, identical input data were used. Packet arrivals were simulated using a Poisson process ^43^ with exponential inter-arrival times:
  - Generate a uniform random variable $\:\mathrm{y}\in\:\left[\mathrm{0,1}\right]$.
  - Compute inter-arrival time:
    15 $$\:\boldsymbol{I}\boldsymbol{n}\boldsymbol{t}\boldsymbol{e}\boldsymbol{r}-\boldsymbol{a}\boldsymbol{r}\boldsymbol{r}\boldsymbol{i}\boldsymbol{v}\boldsymbol{a}\boldsymbol{l}\:\boldsymbol{T}\boldsymbol{i}\boldsymbol{m}\boldsymbol{e}=-\frac{1}{\boldsymbol{\lambda\:}}\mathbf{ln}(1-\boldsymbol{y})$$
    where: $\:\lambda\:$ is the packet arrival rate (packets per second).
    y is the random generated number between 0 and 1.
    The use of 1- y avoids ln (0).
    The negative sign ensures positive inter-arrival times.
  - Update packet arrival time; starting from an initial time (usually 0).
    16 $${\rm{New Time = Old Time + Inter - arrival Time }}$$
    To maintain a consistent data rate at the BS, the non-dead nodes are required to transmit additional packets to compensate for the deficit caused by node failures.
5. Packet aggregation: To minimize redundant data, improve energy efficiency, reduce network congestion, and enhance data processing ^44^, packet aggregation was applied.
  Aggregation can be based on either a fixed number of packets or a fixed time interval:
  - Packet-count-based aggregation; ensures a fixed number of packets are combined, optimizing data utilization, but may introduce delays if packet generation is low.
  - Time-interval aggregation; provides predictable delays, aiding synchronization, but intervals that are too short may result in insufficient packets, while too long intervals increase overall delay.
    In summary, packet-count-based aggregation is more appropriate when the packet generation rate is stable and predictable, whereas time-based aggregation is preferable in scenarios with variable traffic rates; therefore, the latter was adopted in our simulation.
6. Unified simulation algorithm: A single algorithm was developed to track packet generation and transmission from sensor nodes to their cluster heads (CHs), aggregation by CHs, and forwarding to the BS. It also manages BS commands for cluster formation. Of course; the clustering process itself differs depending on the applied method (Energy-Driven K-Means-based LEACH, K-Means-Based LEACH or DEEC-KM). To ensure fair comparisons, the same packet file and network topology were applied consistently across both protocols in every simulation run. Accordingly, both packet information (packet number, source node, generation time) and network topology were generated separately from the simulation algorithm and used identically for both protocols. A comprehensive description of the simulation program is available in ^10^.

### Performance metrics

The evaluation of the proposed Energy-Driven K-Means-based LEACH, traditional K-Means-Based LEACH and DEEC-KM routing protocols was conducted across multiple deployment areas, each incorporating $\:N$ non-uniformly distributed sensor nodes. For each deployment area, a variety of network topologies were considered, and multiple randomly generated packet files $\:P$ were utilized. The following metrics were employed to assess the performance of the wireless sensor networks under the three routing protocols.

#### Network lifetime across the full operational period

To thoroughly assess the longevity and operational reliability of the proposed protocol, the network lifetime was evaluated across its full operational period by tracking the number of nodes remaining alive in each round until the network became fully depleted. Note that the network lifetime directly influences all other performance metrics.

#### Network lifetime at different node death stages

To further assess the longevity and operational reliability of the proposed protocol, network lifetime was examined at several key node-death milestones. This analysis offers deeper insight into how energy depletion progresses over time within the network.

- First node death time (FND).

The FND metric quantifies the elapsed time until the first sensor node exhausts its energy and becomes non-operational. It marks the beginning of network instability and is commonly used to assess the initial stability and early reliability of a routing protocol.

b. Half node death time (HND)/50% node death time.

The HND metric measures the elapsed time until 50% of the total sensor nodes have depleted their energy. It provides insight into the duration for which the network remains partially operational and reflects the sustained performance of the routing protocol during the mid-life stage.

c. Last node death time (LND).

The LND metric captures the elapsed time until the final sensor node in the network depletes its energy, signifying the complete failure of the network. This metric represents the maximum achievable network lifetime and highlights the endurance provided by the routing protocol.

#### Improvement percentage in network lifetime

To quantify improvements, the network lifetime achieved by the proposed Energy-Driven K-Means-based LEACH protocol was compared against the traditional K-Means-based LEACH protocol across all deployment areas, topologies, and packet-generation files. The percentage improvement in network lifetime was calculated using the following expression:

17 $$\:{\boldsymbol{I}\boldsymbol{m}\boldsymbol{p}\boldsymbol{r}\boldsymbol{o}\boldsymbol{v}\boldsymbol{e}\boldsymbol{m}\boldsymbol{e}\boldsymbol{n}\boldsymbol{t}\:\boldsymbol{P}\boldsymbol{e}\boldsymbol{r}\boldsymbol{c}\boldsymbol{e}\boldsymbol{n}\boldsymbol{t}\boldsymbol{a}\boldsymbol{g}\boldsymbol{e}\:\boldsymbol{i}\boldsymbol{n}\:\boldsymbol{N}\boldsymbol{e}\boldsymbol{t}\boldsymbol{w}\boldsymbol{o}\boldsymbol{r}\boldsymbol{k}\:\boldsymbol{L}\boldsymbol{i}\boldsymbol{f}\boldsymbol{e}\boldsymbol{t}\boldsymbol{i}\boldsymbol{m}\boldsymbol{e}}_{\boldsymbol{n},\:\boldsymbol{p}}=\frac{{\left({NDT}_{EAKL}\right)}_{n,p}}{{\left({NDT}_{KL}\right)}_{n,p}}\boldsymbol{*}100$$

where: $\:{Improvement\:Percentage\:in\:Network\:Lifetime}_{n,\:p}$denotes the percentage improvement in the lifetime of network $\:n$ for the randomly generated packet file *p*, achieved using the proposed Energy-Driven K-Means-based LEACH routing protocol compared to the traditional K-Means-Based LEACH protocol.

$\:{\left({NDT}_{EAKL}\right)}_{n,p}$ is the network death time under the proposed Energy-Driven K-Means-based LEACH protocol.

$\:{\left({NDT}_{KL}\right)}_{n,p}$ is the network death time under the traditional K-Means-Based LEACH protocol.

$\:n\in\:Topology\:N$ represents the non-uniformly distributed network topology.

$\:p\in\:Packet\:files\:P$ represents the randomly generated packet file.

For each network , the following metrics were derived:

$$\:\boldsymbol{M}\boldsymbol{a}\boldsymbol{x}\:{\left(\boldsymbol{I}\boldsymbol{m}\boldsymbol{p}\boldsymbol{r}\boldsymbol{o}\boldsymbol{v}\boldsymbol{e}\boldsymbol{m}\boldsymbol{e}\boldsymbol{n}\boldsymbol{t}\:\boldsymbol{P}\boldsymbol{e}\boldsymbol{r}\boldsymbol{c}\boldsymbol{e}\boldsymbol{n}\boldsymbol{t}\boldsymbol{a}\boldsymbol{g}\boldsymbol{e}\:\boldsymbol{i}\boldsymbol{n}\:\boldsymbol{N}\boldsymbol{e}\boldsymbol{t}\boldsymbol{w}\boldsymbol{o}\boldsymbol{r}\boldsymbol{k}\:\boldsymbol{L}\boldsymbol{i}\boldsymbol{f}\boldsymbol{e}\boldsymbol{t}\boldsymbol{i}\boldsymbol{m}\boldsymbol{e}\right)}_{\boldsymbol{n}}=\boldsymbol{m}\boldsymbol{a}\boldsymbol{x}\:\left({\boldsymbol{I}\boldsymbol{m}\boldsymbol{p}\boldsymbol{r}\boldsymbol{o}\boldsymbol{v}\boldsymbol{e}\boldsymbol{m}\boldsymbol{e}\boldsymbol{n}\boldsymbol{t}\:\boldsymbol{P}\boldsymbol{e}\boldsymbol{r}\boldsymbol{c}\boldsymbol{e}\boldsymbol{n}\boldsymbol{t}\boldsymbol{a}\boldsymbol{g}\boldsymbol{e}\:\boldsymbol{i}\boldsymbol{n}\:\boldsymbol{N}\boldsymbol{e}\boldsymbol{t}\boldsymbol{w}\boldsymbol{o}\boldsymbol{r}\boldsymbol{k}\:\boldsymbol{L}\boldsymbol{i}\boldsymbol{f}\boldsymbol{e}\boldsymbol{t}\boldsymbol{i}\boldsymbol{m}\boldsymbol{e}}_{\boldsymbol{n},\:\boldsymbol{p}}\right)$$

$$\:\boldsymbol{M}\boldsymbol{i}\boldsymbol{n}\:{\left(\boldsymbol{I}\boldsymbol{m}\boldsymbol{p}\boldsymbol{r}\boldsymbol{o}\boldsymbol{v}\boldsymbol{e}\boldsymbol{m}\boldsymbol{e}\boldsymbol{n}\boldsymbol{t}\:\boldsymbol{P}\boldsymbol{e}\boldsymbol{r}\boldsymbol{c}\boldsymbol{e}\boldsymbol{n}\boldsymbol{t}\boldsymbol{a}\boldsymbol{g}\boldsymbol{e}\:\boldsymbol{i}\boldsymbol{n}\:\boldsymbol{N}\boldsymbol{e}\boldsymbol{t}\boldsymbol{w}\boldsymbol{o}\boldsymbol{r}\boldsymbol{k}\:\boldsymbol{L}\boldsymbol{i}\boldsymbol{f}\boldsymbol{e}\boldsymbol{t}\boldsymbol{i}\boldsymbol{m}\boldsymbol{e}\right)}_{\boldsymbol{n}}=\boldsymbol{m}\boldsymbol{i}\boldsymbol{n}\:\left({\boldsymbol{I}\boldsymbol{m}\boldsymbol{p}\boldsymbol{r}\boldsymbol{o}\boldsymbol{v}\boldsymbol{e}\boldsymbol{m}\boldsymbol{e}\boldsymbol{n}\boldsymbol{t}\:\boldsymbol{P}\boldsymbol{e}\boldsymbol{r}\boldsymbol{c}\boldsymbol{e}\boldsymbol{n}\boldsymbol{t}\boldsymbol{a}\boldsymbol{g}\boldsymbol{e}\:\boldsymbol{i}\boldsymbol{n}\:\boldsymbol{N}\boldsymbol{e}\boldsymbol{t}\boldsymbol{w}\boldsymbol{o}\boldsymbol{r}\boldsymbol{k}\:\boldsymbol{L}\boldsymbol{i}\boldsymbol{f}\boldsymbol{e}\boldsymbol{t}\boldsymbol{i}\boldsymbol{m}\boldsymbol{e}}_{\boldsymbol{n},\:\boldsymbol{p}}\right)$$

18 $$\:\boldsymbol{A}\boldsymbol{v}\boldsymbol{e}\boldsymbol{r}\:{\left(\boldsymbol{I}\boldsymbol{m}\boldsymbol{p}\boldsymbol{r}\boldsymbol{o}\boldsymbol{v}\boldsymbol{e}\boldsymbol{m}\boldsymbol{e}\boldsymbol{n}\boldsymbol{t}\:\boldsymbol{P}\boldsymbol{e}\boldsymbol{r}\boldsymbol{c}\boldsymbol{e}\boldsymbol{n}\boldsymbol{t}\boldsymbol{a}\boldsymbol{g}\boldsymbol{e}\:\boldsymbol{i}\boldsymbol{n}\:\boldsymbol{N}\boldsymbol{e}\boldsymbol{t}\boldsymbol{w}\boldsymbol{o}\boldsymbol{r}\boldsymbol{k}\:\boldsymbol{L}\boldsymbol{i}\boldsymbol{f}\boldsymbol{e}\boldsymbol{t}\boldsymbol{i}\boldsymbol{m}\boldsymbol{e}\right)}_{\boldsymbol{n}}=\frac{1}{\boldsymbol{P}}\:\sum\:\left({\boldsymbol{I}\boldsymbol{m}\boldsymbol{p}\boldsymbol{r}\boldsymbol{o}\boldsymbol{v}\boldsymbol{e}\boldsymbol{m}\boldsymbol{e}\boldsymbol{n}\boldsymbol{t}\:\boldsymbol{P}\boldsymbol{e}\boldsymbol{r}\boldsymbol{c}\boldsymbol{e}\boldsymbol{n}\boldsymbol{t}\boldsymbol{a}\boldsymbol{g}\boldsymbol{e}\:\boldsymbol{i}\boldsymbol{n}\:\boldsymbol{N}\boldsymbol{e}\boldsymbol{t}\boldsymbol{w}\boldsymbol{o}\boldsymbol{r}\boldsymbol{k}\:\boldsymbol{L}\boldsymbol{i}\boldsymbol{f}\boldsymbol{e}\boldsymbol{t}\boldsymbol{i}\boldsymbol{m}\boldsymbol{e}}_{\boldsymbol{n},\:\boldsymbol{p}}\right)$$

where: $\:{Max,\:\:Min\:\&\:Aver\:\left(Improvement\:Percentage\:in\:Network\:Lifetime\right)}_{n}$represent, respectively, the maximum, minimum, and average percentage improvement in the lifetime of network $\:n$ achieved using the proposed Energy-Driven K-means-based LEACH routing protocol compared to using the traditional K-Means-Based LEACH protocol computed across all randomly generated packet files $\:p\in\:Packet\:files\:P$.

#### Overall energy consumption across the full operation period

This metric measures the cumulative energy consumed by all nodes from the start of operation up to each round, continuing until the network is fully depleted. By summing the energy used up to each round, it provides a view of the network’s energy consumption over time (rounds), allowing for an assessment of how efficiently the proposed protocol utilizes node energy resources.

#### Number of delivered packets

The number of delivered packets is the total count of data packets successfully received at the BS during the entire operational lifetime of the network. It reflects how much useful information the WSN is able to deliver before the nodes deplete their energy. This metric captures the packet generation efficiency (i.e., how long nodes stayed alive and sending data), and the routing efficiency (i.e., how effectively the protocol forwards packets without excessive energy waste or premature node death). Thus, a higher value indicates a more energy-efficient and longer-lasting routing protocol.

#### Number of better-performing packets’ files

For each deployment area and topology, the number of packet files yielding superior performance was recorded for both the proposed Energy-Driven K-means-based LEACH and the traditional K-Means-Based LEACH routing protocols. In cases where the difference in network lifetime was less than 0.5%, or when each protocol performed comparably across packet files, the performance was considered equivalent.

### Simulation results

Simulation parameters for protocol comparison are summarized in Table 2. All parameters, including energy values, control and data packet sizes, and related settings, were adopted from^45^. Simulations were conducted over nine deployment areas ranging from $\:500\:\times\:500\:{m}^{2\:}$($\:250,000\:{m}^{2\:})$ to $\:100\:\times\:100\:{m}^{2\:}$($\:10,000\:{m}^{2\:}$). The base station (BS) was centrally located within each network area. For each deployment area, ten non-uniformly distributed node topologies were generated, and thirty randomly generated packet files were tested for each topology.

For each deployment area, the average percentage of node-to-node distances below the threshold $\:{d}_{O}$ was calculated across the ten topologies. This metric, denoted as $\:APDN$, along with the density of random network nodes ($\:DRNN$), and the deployment area are presented in Table 3. The *DRNN* was calculated using the smallest deployment area of $\:100\:\times\:100\:{m}^{2\:}$as the unit area. Across nine deployment areas and ten topologies each, a total of 90 cases were evaluated using thirty random packet files.

**Table 2:** Simulation parameters.

Description	Value
Number of sensor nodes in the network	100 nodes
Packets’ rate	100 packets/s
Initial-energy of each node in the network	3 Joules
Electronic energy ($\:{E}_{elec}$)	50 nJ/bit
Amplification energy ($\:{E}_{amp}$) for the multipath model	0.0013 pJ/bit/m^4^
Amplification energy ($\:{E}_{fs}$) for the free-space model	10 pJ/bit/m^2^
Aggregation energy	5 nJ/bit
Control packet size	200 bits
Data packet size	6400 bits
Cluster update interval	5 s
Transmission time	0.01

**Table 3:** Network models and their corresponding topology characteristics.

Area	500 × 500	400 × 400	350 × 350	300 × 300	250 × 250	200 × 200	150 × 150	125 × 125	100 × 100
APDN	10.4	14.7	19	23.8	31.3	45.2	62.7	74.6	94.6
DRNN	4	6.25	8.16	11.11	16	25	44.44	64	100

It is evident from Table 3 that as the deployment area decreases, the density of randomly distributed nodes ($\:DRNN$) increases, which is accompanied by a corresponding rise in the average percentage of distances below the threshold ($\:APDN$). This indicates that in smaller areas, nodes are closer to each other on average, resulting in shorter inter-node distances. For clarity and brevity in the subsequent sections, the discussion will primarily reference the node density ($\:DRNN$) as a representative indicator of network spatial characteristics, without repeatedly mentioning either the deployment area or the $\:APDN$. Note that the full details of all three factors are provided in the captions of each figure, where each subfigure (a)–(i) corresponds to one of the nine deployment areas.

#### Network lifetime across the full operational period

**Fig. 6:**
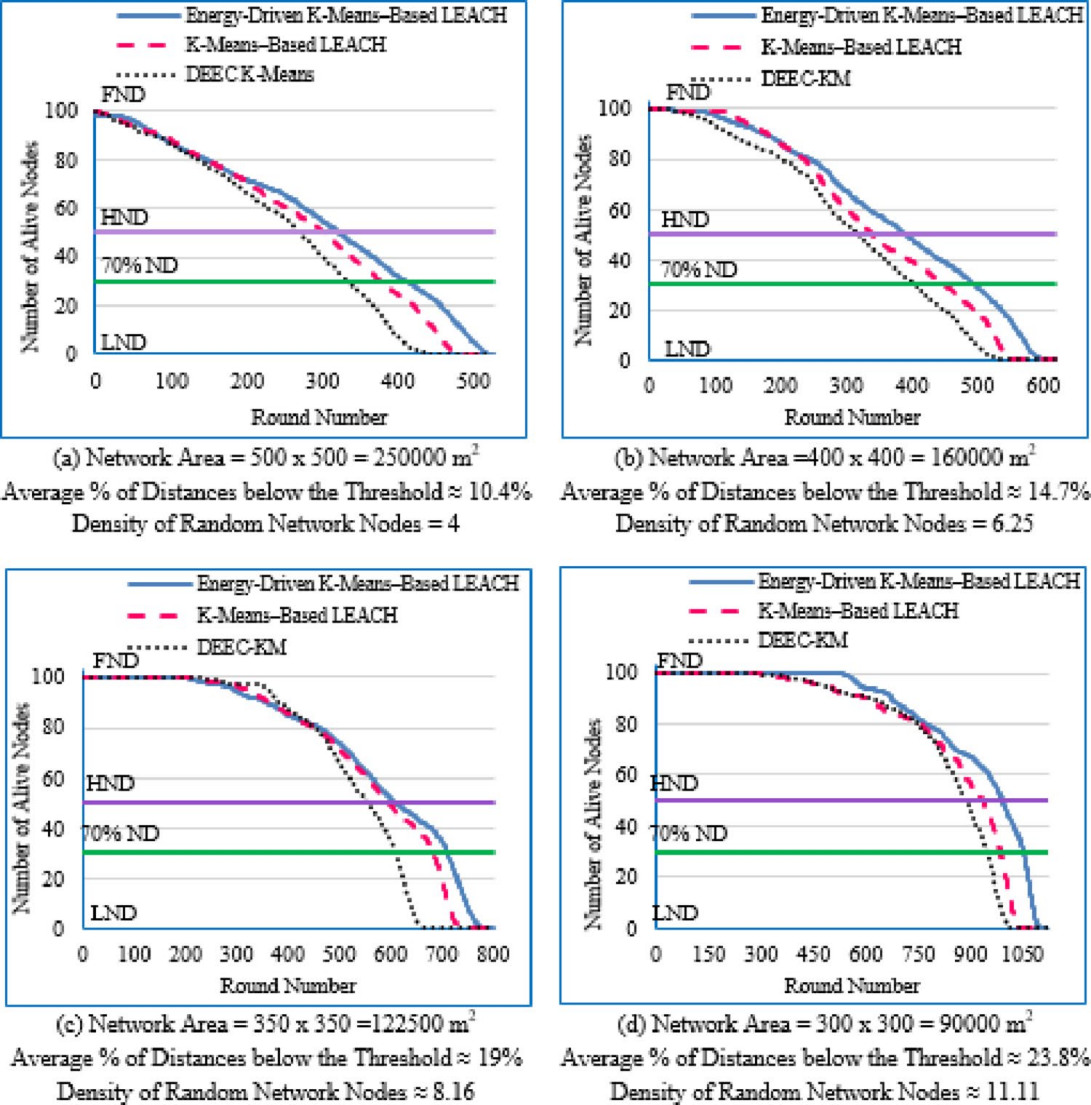

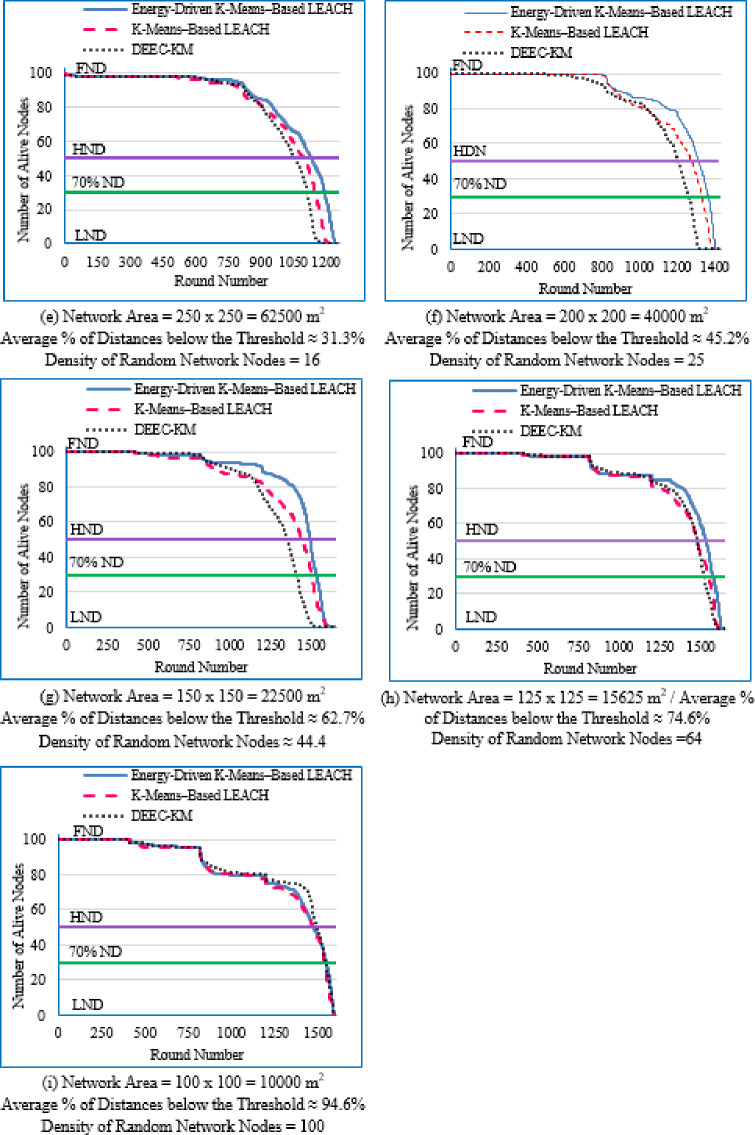
Network lifetime across the full operational period of the three routing protocols across the nine network sizes listed in Table 3, evaluated over ten topologies and thirty randomly generated packet files per topology.

Figure 6 illustrates the average network lifetime evaluated across the nine deployment areas for the proposed Energy-Driven K-means-based LEACH, the traditional K-Means-Based LEACH, and the DEEC-KM routing protocols.

For each graph in Fig. 6, the **FND** corresponds to the point at which 99 nodes (N–1) are still alive, which is visually aligned with the upper horizontal line in the plot. The **HND** occurs when 50 nodes (N/2) remain alive and is indicated by the purple horizontal line. The **LND** corresponds to zero alive nodes, which aligns with the lower horizontal line in the plot area. Although network lifetime was measured from full node availability to the death of the last node, in practical scenarios a WSN is considered non-functional once a predefined percentage of nodes have failed, typically between 70% and 75%. Therefore, a horizontal (green) line indicating this threshold (set here at 70% node death^13^ has been added to each figure.

From Fig. 6, the following observations can be made:

- Low-density networks (4 and 6.25 nodes/unit area): The proposed Energy-Driven K-Means-based LEACH shows the slowest decline in alive nodes and clearly outperforms both baselines. It reaches the FND line (upper line), the HND line (purple line), the 70% ND line (green line), and the LND (bottom line) significantly later than the other two protocols, demonstrating superior stability across all network stages. DEEC-KM reaches all four markers earliest and intersects the LND line (bottom line) far sooner due to unbalanced clusters and inefficient energy usage. Traditional K-Means-based LEACH performs better than DEEC-KM but still reaches the HND line (purple line), the 70% ND line (green line), and the LND (bottom line) indicators before the proposed protocol. It is observed that in certain cases traditional K-Means-based LEACH temporarily outperforms the proposed protocol during the initial phase of network operation, which is evident from its slightly delayed intersection with the FND line.
- Medium-density networks (8.16 to 25 nodes/unit area): The proposed protocol maintains a clear lead across all lifetime indicators. It consistently delays reaching the FND, HND, 70% ND and LND lines compared with both baselines. DEEC-KM intersects the FND, HND and 70% ND lines very early and approaches the LND line much sooner, while K-Means-based LEACH remains consistently between the two. Although the performance gap narrows somewhat as density increases, the proposed method still preserves a visibly larger separation from all ND reference lines, illustrating the effectiveness of its distance-sensitive energy model. However; similarly to the behavior observed in low-density networks, there are cases where the traditional K-means-based LEACH briefly outperforms the proposed protocol during the early stage of operation, as indicated by its slightly delayed intersection with the FND line.
- High-density networks (44.44 to 100 nodes/unit area): The ordering of the protocols remains unchanged across all lifetime markers: the proposed protocol reaches the FND, HND, 70% ND, and LND lines last, followed by K-Means-based LEACH, while DEEC-KM performs the worst. However; the performance of the proposed Energy-Driven K-means-based LEACH and the traditional K-Means-Based LEACH is comparable, in fact in certain low-density scenarios, the proposed protocol may exhibit a slightly earlier first node death. At very high densities—especially at 100 × 100; the curves of all three protocols intersect the ND lines (including the 70% ND threshold) at nearly the same points, indicating that when all inter-node distances become uniformly short, protocol differences diminish dramatically.

This can be justified as follows:

- The proposed Energy-Driven K-Means-based LEACH protocol consistently provides the best performance because of its adaptive switching between the $\:{d}^{2}$ and the $\:{d}^{4}$ energy models (based on the actual distribution of inter-node distances) reduces unnecessary energy consumption. This leads to more balanced clusters, more efficient transmission paths, and protection of nodes near the BS from early depletion.
- DEEC-KM performs the worst across all densities. Although it uses K-means for cluster creation, it selects CHs solely based on residual energy, ignoring transmission distance—despite distance being the dominant factor in the radio energy dissipation model. As a result, CH selection becomes energetically-imbalanced and inefficient. In contrast, the other two protocols (K-Means-based-LEACH and the proposed Energy-Driven K-Means-based LEACH) incorporate distance information (directly or indirectly) leading to more energy-efficient CH selection.
- While K-Means-based LEACH consistently outperforms DEEC-KM, it remains inferior to the proposed protocol. This is mainly because it relies solely on geometric clustering without integrating any energy-aware mechanism, which leads to suboptimal CH placement and faster node depletion compared with the proposed Energy-Driven approach.
- During the initial rounds, all nodes still have nearly equal residual energy, and energy depletion has not yet begun to influence clustering decisions significantly. In this phase, traditional K-Means-based LEACH benefits from purely geometric clustering, which naturally produces compact clusters with short intra-cluster distances; especially in dense or uniformly distributed networks. Because transmission energy is still low at the beginning and the network is fully connected, these short distances allow K-means-based LEACH to achieve slightly lower per-round energy consumption during the very early stage. In contrast, the proposed Energy-Driven K-Means-based LEACH applies its adaptive distance penalization (switching between $\:{d}^{2}$ and the $\:{d}^{4}$). While this mechanism becomes highly beneficial as distances grow or as the network becomes irregular, it may introduce slightly higher initial overhead compared with the purely geometric version. This overhead is negligible in the long run but can result in a small delay in reaching the FND, making traditional K-means-based LEACH appear momentarily superior at the very beginning.

However, once nodes begin to deplete energy and long-distance transmissions increasingly dominate the communication cost, the geometric clustering of K-Means-based LEACH becomes insufficient. The proposed protocol then consistently outperforms it due to its energy-aware adjustments, which better reflect the radio dissipation model.

#### Network lifetime at different node death stages

As shown in Fig. 6; which presents the average network lifetime of the three routing protocols across the nine network sizes; there are instances where traditional K-Means-based LEACH briefly outperforms the proposed Energy-Driven K-Means-based LEACH during the very early stage of operation. This behavior, reflected by its slightly delayed intersection with the FND line, which also appears in very dense network deployments. To better capture and analyze these cases, the FND, HND, and LND times were measured for both traditional K-Means-based LEACH and the proposed Energy-Driven protocols across all nine network sizes, using ten topologies and thirty randomly generated packet files per topology. For clarity, each graph represents the performance of the two protocols for a single area, showing results for each topology independently within that specific area.

Although such reversals may occur between the two K-means-based protocols, no cases were observed in which DEEC-KM outperformed the proposed protocol. The Energy-Driven K-Means-based LEACH consistently reaches the FND, HND, 70% ND, and LND points earlier than DEEC-KM across all densities. For completeness, the same parameters (FND, HND, and LND) were also measured for DEEC-KM for each area and for each topology individually, and in all scenarios DEEC-KM remained inferior.

To avoid unnecessary complexity in the charts and because the detailed FND, HND, and LND behavior of DEEC-KM does not contribute additional insight, DEEC-KM is excluded from the comparative plots. A focused comparison between K-Means-based LEACH and the proposed Energy-Driven K-Means-based LEACH provides a clearer and more informative analysis.

**Fig. 7:**
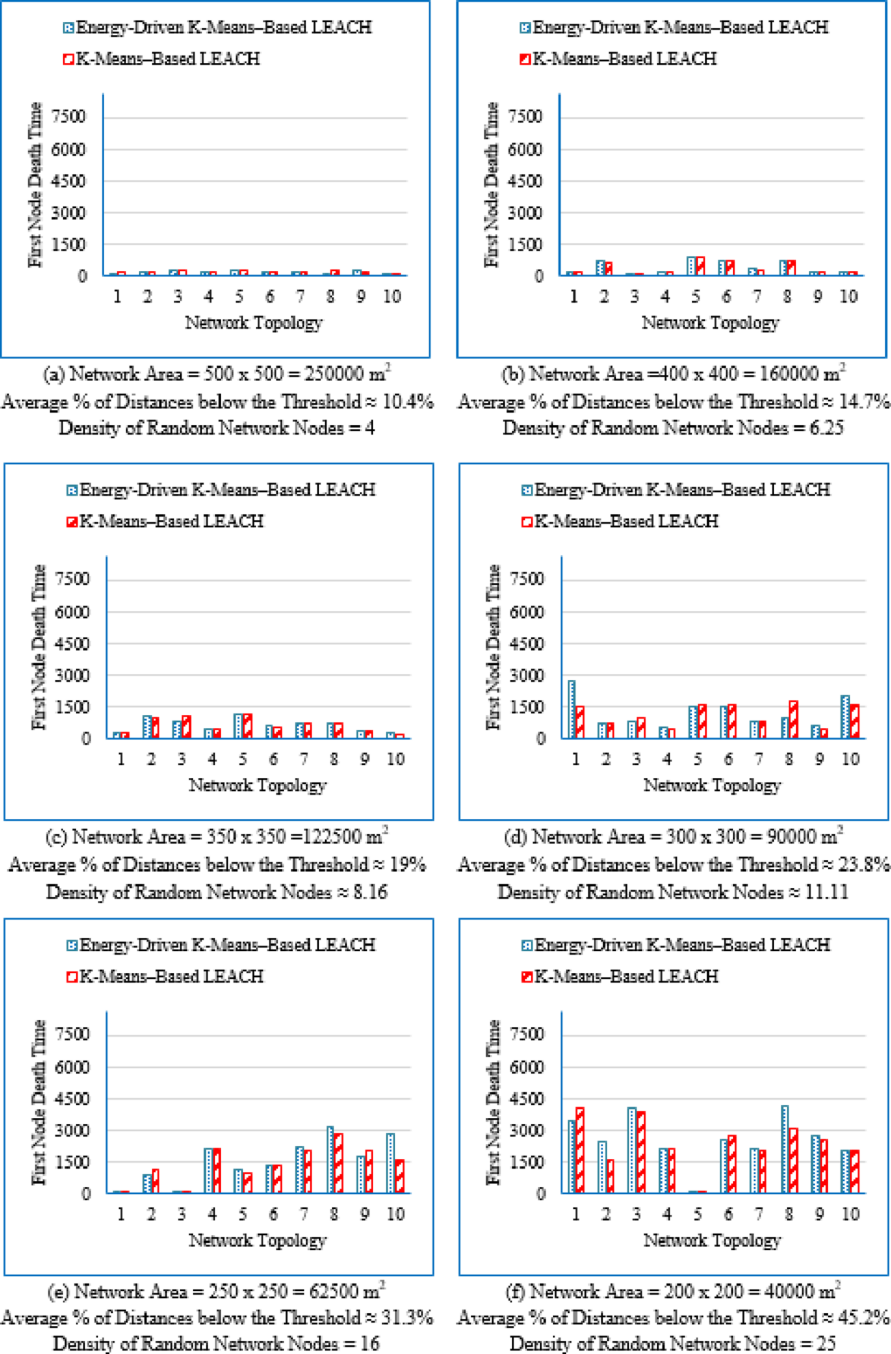

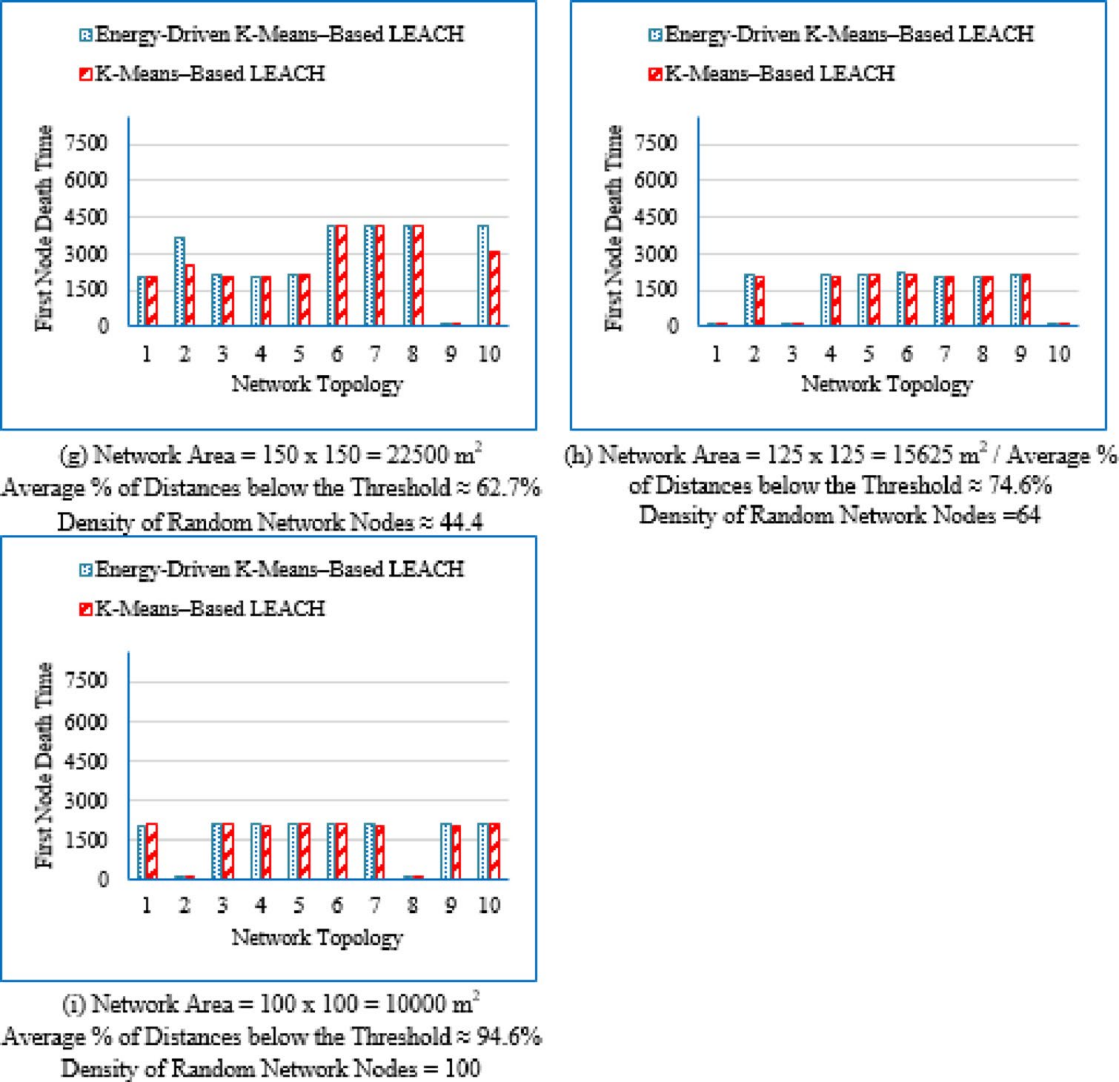
First node death time for the traditional K-Means-Based LEACH and the proposed Energy-Driven K-Means-based LEACH routing protocols across the nine network sizes listed in Table 3, each evaluated using ten topologies and thirty randomly generated packet files per topology.

**Fig. 8:**
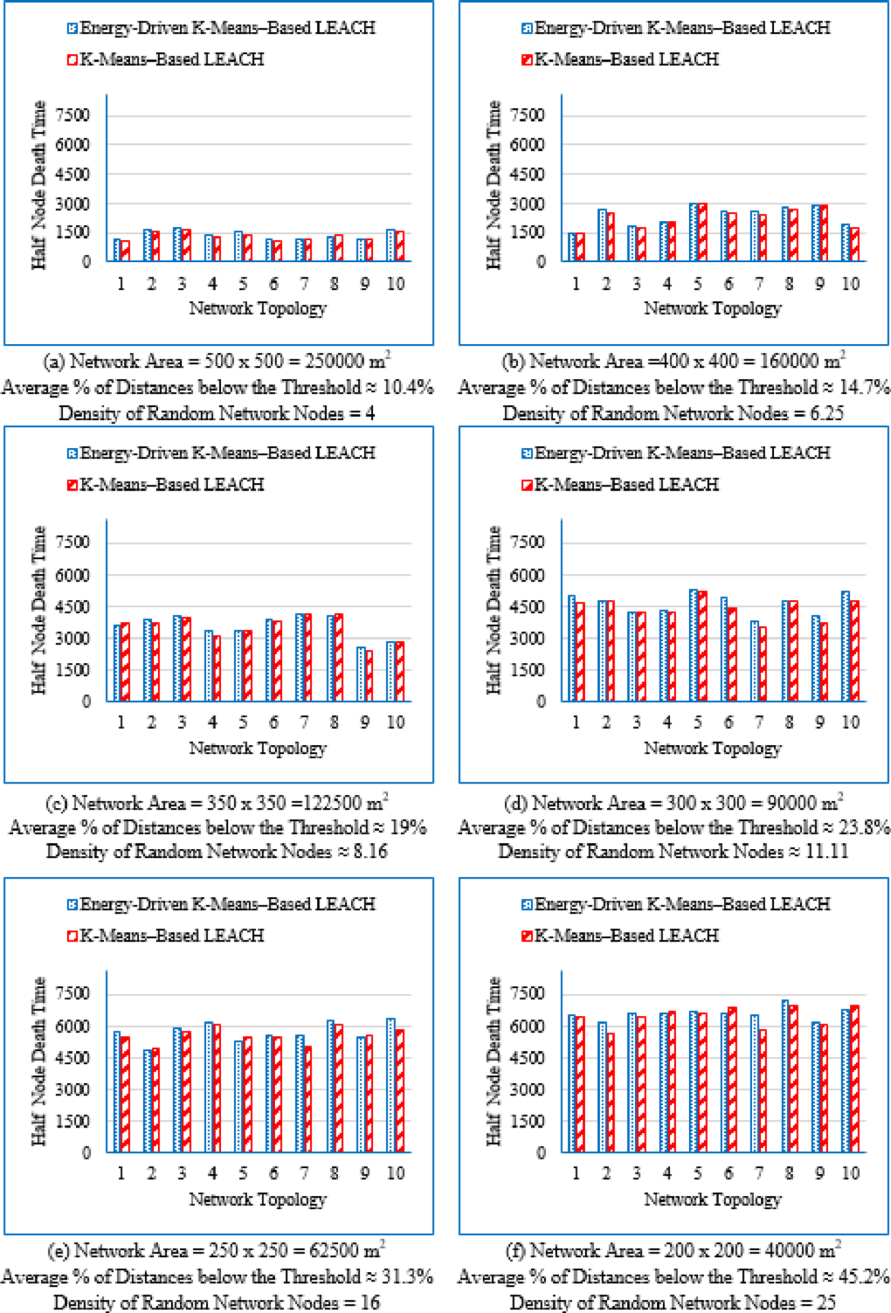

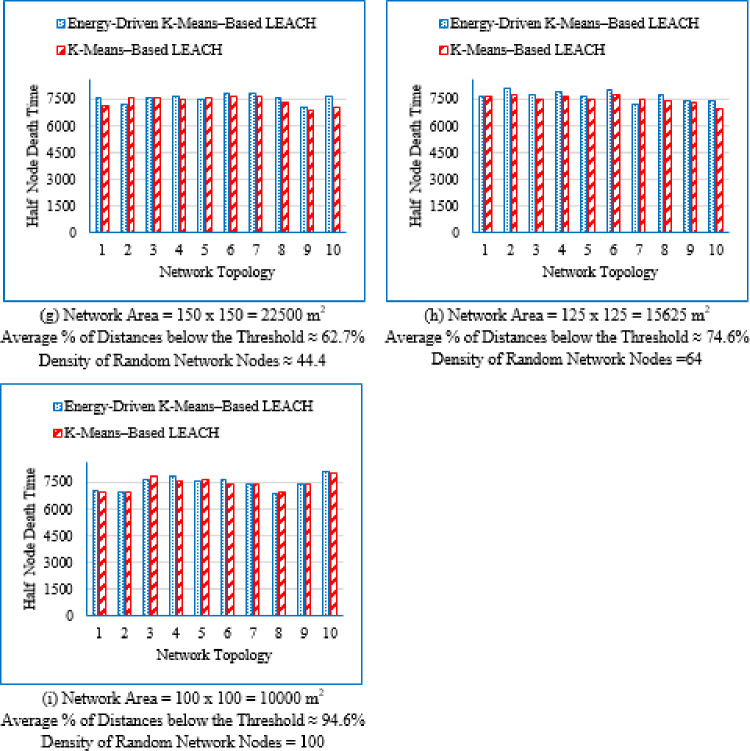
Half node death time for the traditional K-Means-Based LEACH and the proposed Energy-Driven K-Means-Based LEACH routing protocols across the nine network sizes listed in Table 3, each evaluated using ten topologies and thirty randomly generated packet files per topology.

**Fig. 9:**
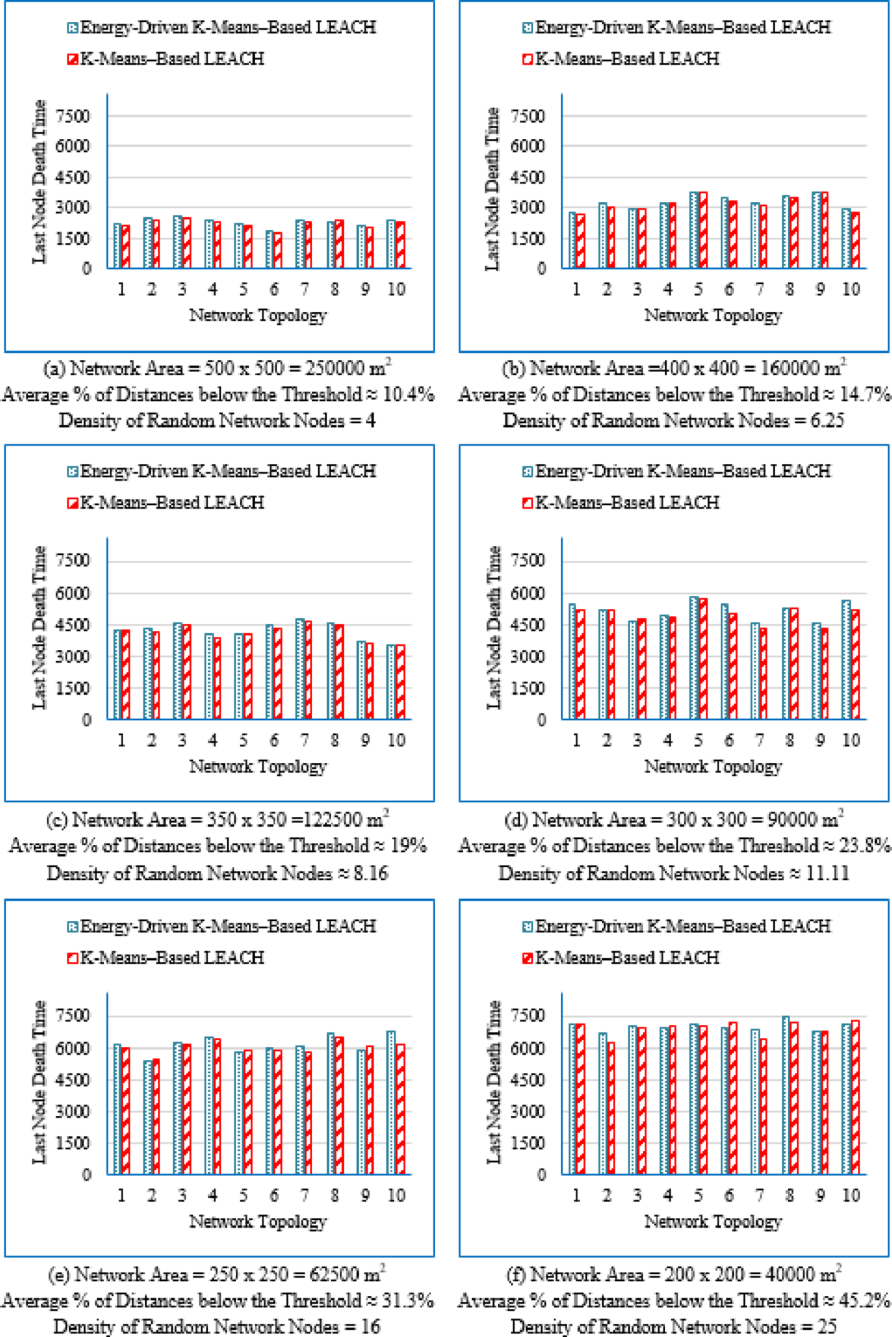

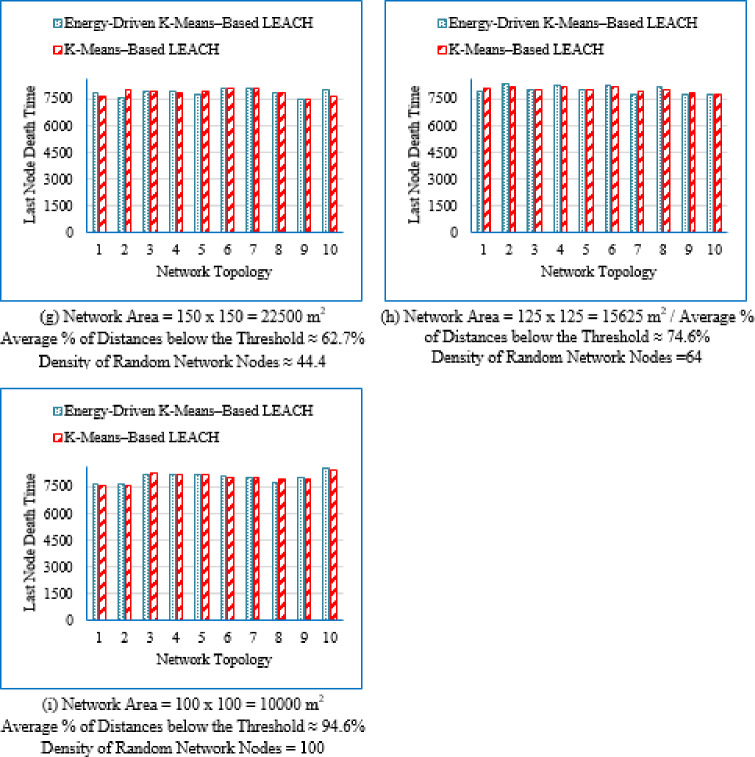
Last Node Death Time for both the traditional K-Means-Based LEACH and the proposed Energy-Driven K-means–based LEACH routing protocols evaluated across the nine network sizes listed in table 3, each with ten topologies and thirty different packet files per topology.

Figures 7, 8 and 9 illustrate FND, HND, and LND across the nine deployment areas for both the proposed Energy-Driven K-means-based LEACH protocol and the traditional K-Means-Based LEACH.

- First node death time (FND).

Figure 7 illustrates that the proposed Energy-Driven K-Means-based LEACH protocol generally extends the time to first node death relative to the K-Means-Based LEACH protocol across most deployment areas. In high-density networks, the performance of both protocols is comparable, while in certain low-density scenarios, the proposed protocol may exhibit a slightly earlier first node death.

These observations indicate that improvements during the initial stage are not universally guaranteed across all topologies; however, the proposed protocol demonstrates superior performance in the majority of cases. Early-stage stability remains a critical consideration, particularly for applications requiring uninterrupted sensing from the onset of network operation.

b. Half node death time (HND).

Figure 8 demonstrates that the proposed protocol more consistently prolongs the stability period until 50% of the sensor nodes have depleted their energy, emphasizing its enhanced energy-balancing capability during the mid-life stage of the network. In contrast, the traditional K-Means-Based LEACH protocol exhibits accelerated node depletion as the network progresses. The proposed method achieves improved performance by maintaining equitable cluster-head assignment, thereby sustaining a larger number of active nodes over an extended period.

c. Last node death time (LND).

Figure 9 shows that the proposed Energy-Driven K-Means-based LEACH protocol generally extends the final survival of the network compared to the traditional K-Means-Based LEACH, particularly in low- and medium-density deployments. This demonstrates its ability to sustain network connectivity for a longer duration toward the end of the network lifetime. However, in highly dense deployments, the performance gap becomes marginal, indicating that end-stage improvements are more strongly topology-dependent than those observed during the mid-life phase.

**Fig. 10:**
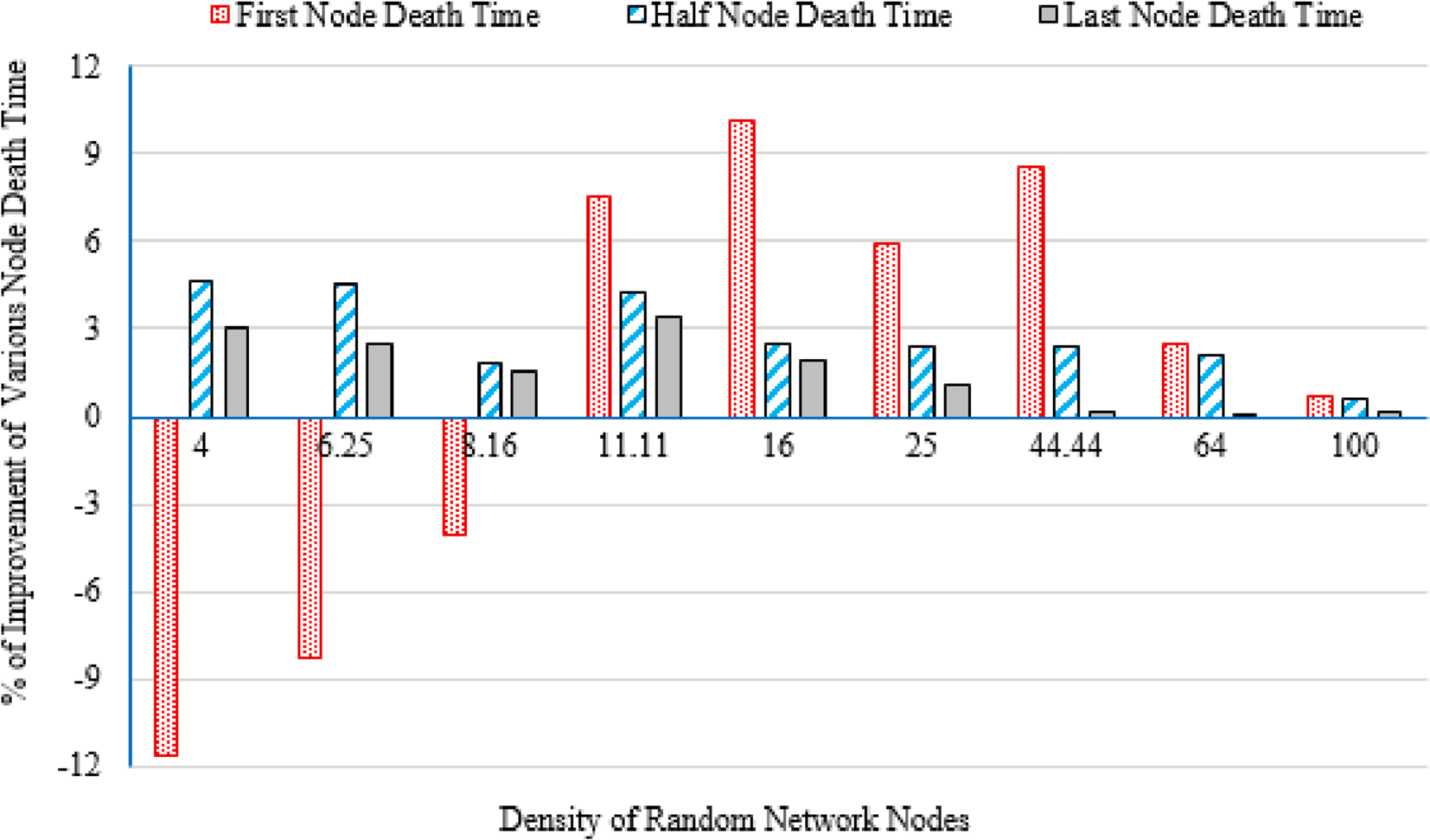
Improvement in First, Half, and Last Node Death Time using the traditional K-Means-Based LEACH and the proposed Energy-Driven K-means-based LEACH routing protocols, across the nine network configurations listed in Table 3.

Figure 10 provides a consolidated view of the results by illustrating the percentage improvement in First Node Death (FND), Half Node Death (HND), and Last Node Death (LND) achieved by the proposed Energy-Driven K-Means-based LEACH protocol compared to the traditional K-Means-Based LEACH across different network densities. Negative values indicate scenarios in which the proposed protocol underperforms relative to the traditional K-Means-Based LEACH.

From Fig. 10, the following observations can be drawn:

- Low-density networks (4 and 6.25 nodes/unit area): The proposed Energy-Driven K-Means-based LEACH protocol exhibits negative improvement in the early lifetime, with the FND even reaching approximately − 11% at density 4, indicating that the first node dies earlier than in the traditional K-Means-Based LEACH. However, both HND and LND show strong positive improvements, demonstrating that while the protocol may allow an early node to die sooner, it achieves substantially better long-term network survivability; effectively trading early stability for extended endurance.
- Medium-density networks (8.16 to 25 nodes/unit area): The proposed protocol consistently outperforms the traditional approach across all three metrics (FND, HND, and LND). The improvement is most pronounced at density 16, where the FND alone approaches nearly + 10%. This density range therefore represents the most favorable and stable operating region for the proposed algorithm.
- High-density networks (44.44 to 100 nodes/unit area): The improvements across FND, HND, and LND converge toward very small values, typically within + 1 to 2%. This indicates that both protocols behave almost identically in densely deployed networks, where naturally short communication distances already make energy dissipation low and traditional clustering highly efficient.

#### Improvement percentage in network lifetime

**Fig. 11:**
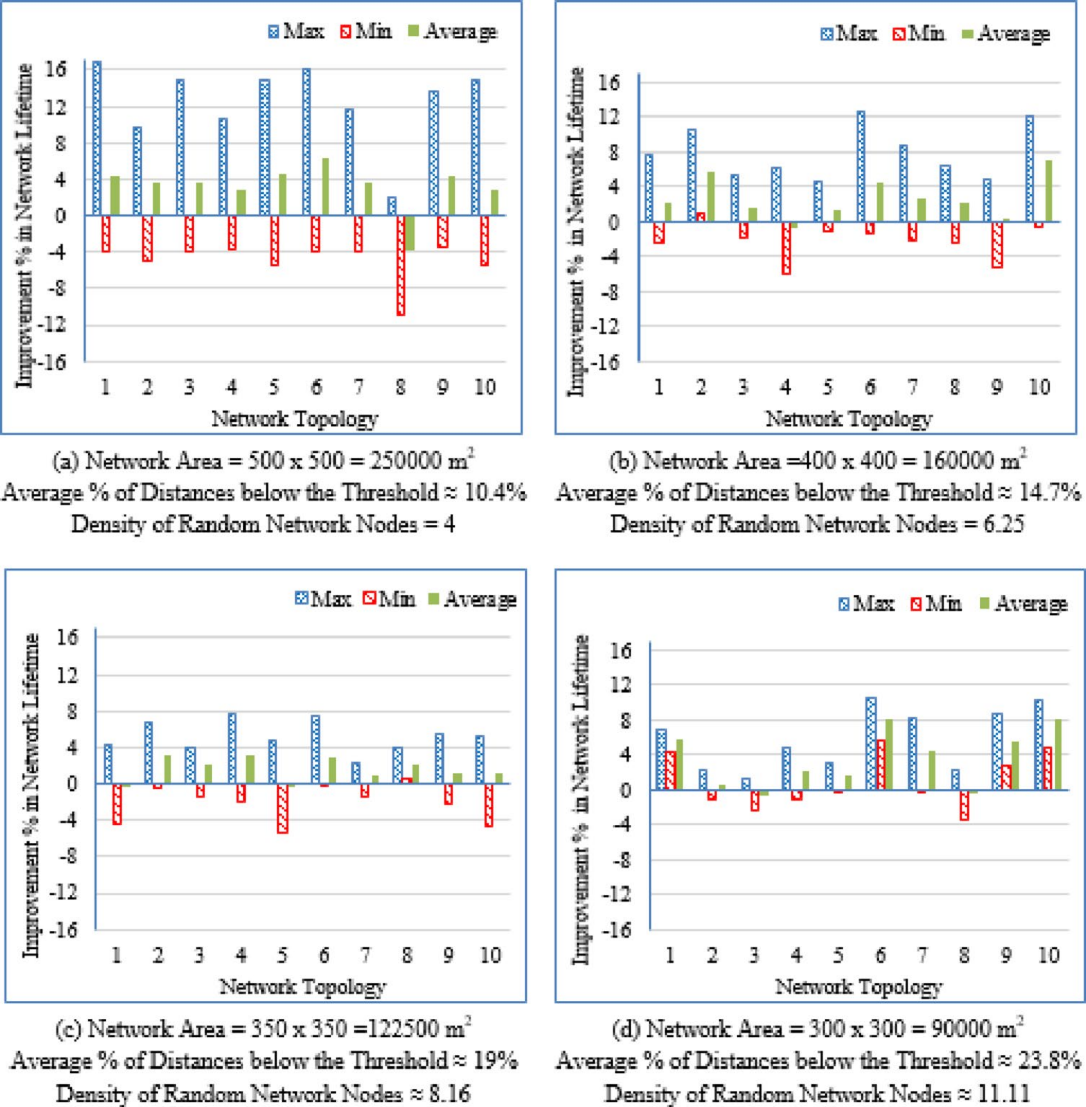

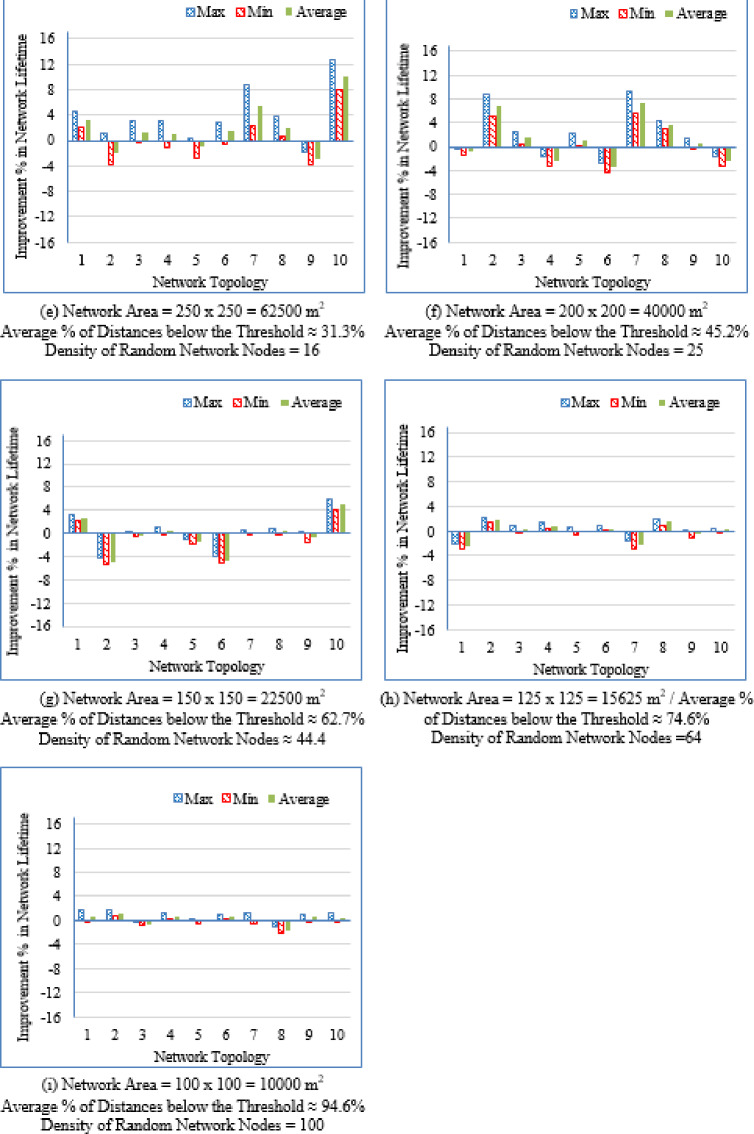
Improvement percentage in network lifetime when applying the proposed Energy-Driven K-means-based LEACH protocol compared to the traditional K-Means-Based LEACH, across the nine node densities listed in Table 3, with ten topologies per density and thirty different packet files per topology.

Figure 11 presents the improvement percentage in network lifetime across the nine network densities   ($\:DRNN$) listed in Table 3, comparing the traditional K-means-based LEACH protocol with the proposed Energy-Driven K-means-based LEACH protocol. For each node density, three metrics are shown: maximum, minimum, and average improvement percentage. Negative values indicate cases where the traditional K-means-based LEACH outperforms the proposed Energy-Driven K-means-based LEACH protocol.

Although the results for DEEC-KM were generated, its network lifetime is always inferior, and its performance trend has already been clearly established in the previous sections. So; the same reasons outlined in Sect. 4.3.2 for excluding them apply here as well.

From Fig. 11, it can be observed that the proposed Energy-Driven K-means-based LEACH protocol achieves significant improvements in network lifetime, particularly in low-density networks. As node density increases, the maximum, minimum, and average improvements gradually decrease, indicating that the performance gain of the proposed protocol diminishes in denser network configurations.

**Fig. 12:**
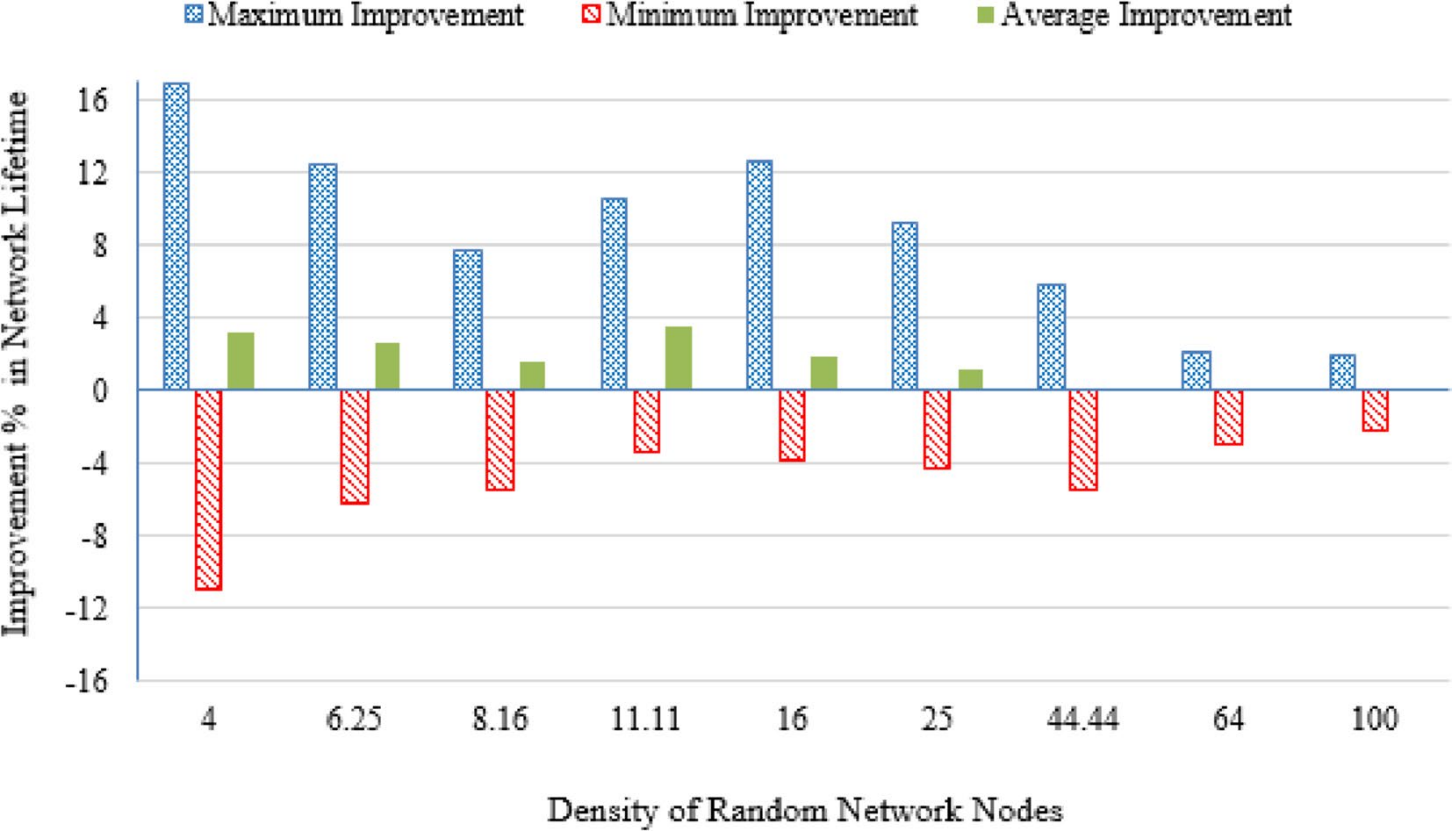
Maximum, minimum, and average improvement percentage in network lifetime using the proposed Energy-Driven K-Means-based LEACH compared to the traditional K-Means-Based LEACH, across the nine network densities listed in Table 3.

Figure 12 summarizes these results by presenting the maximum, minimum, and average improvement percentages in network lifetime achieved by the proposed Energy-Driven K-Means-based LEACH relative to the traditional K-Means-based LEACH. The maximum values represent the scenarios where the proposed protocol significantly outperforms the traditional approach, while negative minimum values indicate cases where the K-Means-Based LEACH performs better.

From Fig. 12, the following observations can be made:

- Maximum percentage improvement: This metric quantifies the extent to which the proposed Energy-Driven K-Means-based LEACH outperforms the K-Means-Based LEACH. The improvement is highest in low-density networks, with maximum gains reaching up to 16.98% for DRNN values of 4 and 6.25 nodes/unit area. As the network becomes denser, the maximum improvement gradually declines, falling below 2% in high-density networks for DRNN = 64 and 100. This confirms that the proposed protocol is particularly effective in large-scale, low-density deployments.
- Minimum percentage improvement: This metric quantifies the extent to which the traditional K-Means-based LEACH outperforms the proposed Energy-Driven K-Means-Based LEACH. Similar to the maximum percentage improvement; the highest value the traditional K-Means-based LEACH prevails is reached at low-density networks (DRNN = 4). As density increases, the minimum gap consistently decreases; indicating that such unfavorable cases become increasingly rare and less significant. Similar to the maximum percentage improvement, the minimum improvement also declines with higher density, falling below 2% in high-density networks (DRNN = 64 and 100).

However, Fig. 11 shows that the extreme minimum value (− 10.96) observed at the lowest density (DRNN = 4) is an isolated outlier resulting from a single topology and therefore does not reflect the general performance trend of the protocol; particularly given that the second-lowest value is already − 5.72. In contrast, the maximum improvement (16.98) observed at the same density is comparable to the highest gains achieved at higher densities (e.g., 12.64, 12.52, 10.6 … for DRNN ≥ 25), indicating that the proposed protocol still demonstrates strong potential even under sparse deployment.

- Average percentage improvement: Across all node densities, the average improvement remains consistently positive, confirming the overall effectiveness of the proposed protocol. However, as node density increases, the average gain gradually declines and becomes negligible, dropping below 1% for higher-density networks (DRNN ≥ 44.44). This indicates that the performance advantage of the proposed protocol becomes marginal under dense deployment conditions.

#### Overall energy consumption across the full operation period

**Fig. 13:**
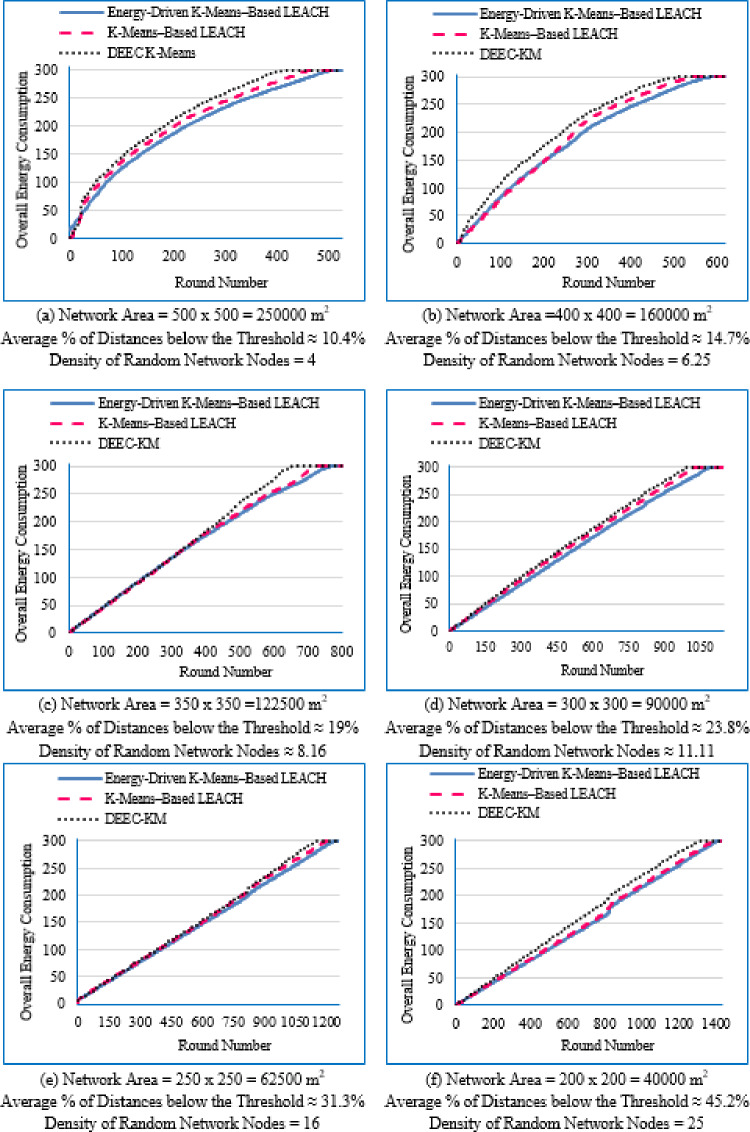

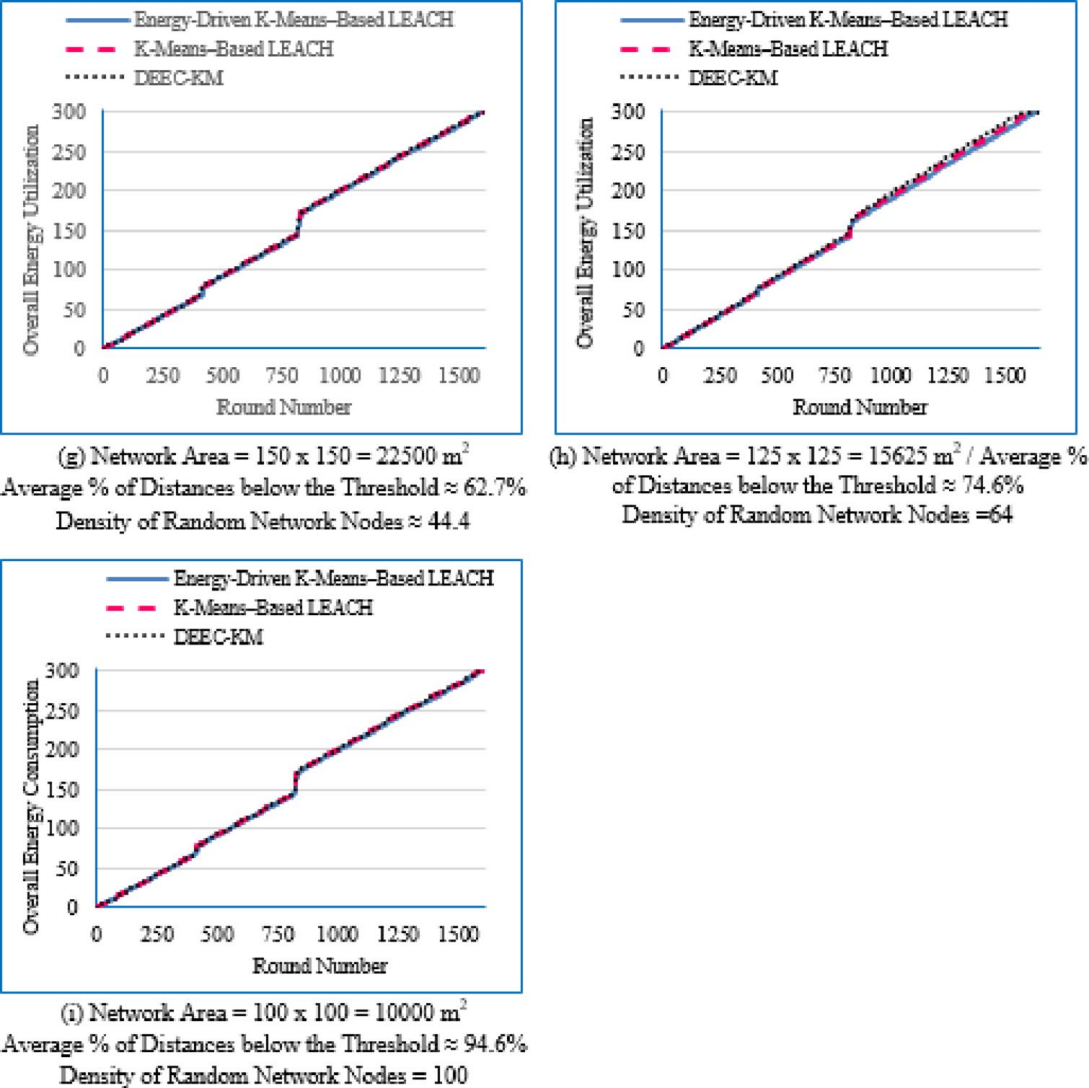
Overall energy consumption for the three protocols routing protocols across the nine network sizes listed in Table 3, each evaluated using ten topologies and thirty randomly generated packet files per topology.

Figure 13 illustrates the average overall energy consumption evaluated across the nine deployment areas for the proposed Energy-Driven K-means-based LEACH, the traditional K-Means-Based LEACH, and the DEEC-KM routing protocols.

From this figure, the following observations can be drawn:

- Low-density networks (4 and 6.25 nodes/unit area): In sparse deployments, the proposed Energy-Driven K-Means-based LEACH protocol consistently shows the lowest overall energy consumption, maintaining a noticeably flatter energy-usage slope across rounds. Traditional K-Means-based LEACH consumes more energy than the proposed method but still performs better than DEEC-KM, which exhibits the highest and fastest-rising energy consumption among the three protocols. This behavior results from the prevalence of long-range multipath transmissions (∝ $\:{d}^{4}$) in sparse networks; the proposed protocol mitigates this cost through its adaptive distance-based energy model, whereas DEEC-KM lacks such a mechanism and wastes more energy in early rounds.
- Medium-density networks (8.16 to 25 nodes/unit area): The proposed protocol continues to be the most energy-efficient, with a consistently lower energy consumption profile across the full operation period. K-Means-based LEACH remains the second best and shows moderate energy savings compared to DEEC-KM. DEEC-KM still consumes the highest amount of energy, although the gap between the three protocols becomes smaller as transmissions increasingly fall within the free-space region (∝ $\:{d}^{2}$). Nevertheless, the proposed method preserves a clear advantage through more balanced clustering and more efficient CH selection, resulting in a slower increase in accumulated energy usage.
- High-density networks (44.44 to 100 nodes/unit area): At higher densities, the proposed protocol remains the least energy-consuming in most scenarios. However, the differences between all three protocols gradually shrink. In very dense settings (particularly at 100 × 100 m^2^); the energy consumption curves of the three protocols become very close, indicating that when inter-node distances are uniformly short, the inherent energy advantages of each scheme diminish. Still, the proposed Energy-Driven K-Means-based LEACH maintains a slight advantage by keeping its consumption curve marginally lower, demonstrating that even in dense deployments, its adaptive energy model continues to prevent unnecessary expenditure.

These results follow the same reasoning presented in “Network lifetime across the full operational period”.

#### Number of delivered packets

**Fig. 14:**
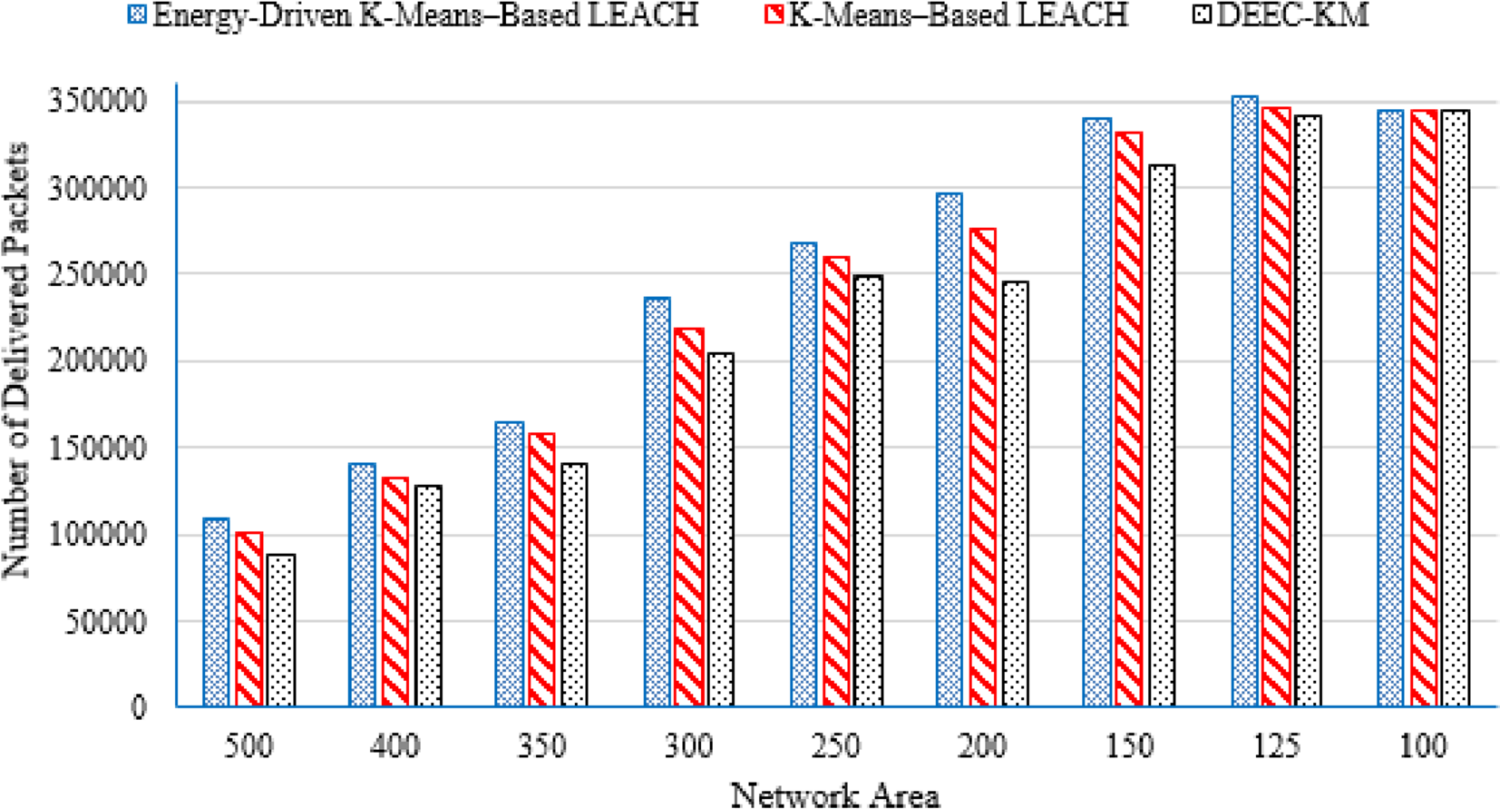
Average number of delivered packets across the nine network densities listed in Table 3 for the three routing protocols; the proposed energy-driven K-means-based LEACH traditional K-means-based LEACH, and the DEEC-KM routing protocols.

Figure 14 illustrates the average number of Delivered Packets evaluated across the nine deployment areas for the proposed Energy-Driven K-means-based LEACH, the traditional K-Means-Based LEACH, and the DEEC-KM routing protocols.

From Fig. 14, the following observations can be made:

- Low-density networks (4 and 6.25 nodes/unit area): The proposed Energy-Driven K-Means-based LEACH achieves the highest number of delivered packets. Traditional K-Means-LEACH performs reasonably well but remains below the proposed method, while DEEC-KM records the lowest delivery count, reflecting its limited capability to sustain node operation under long-distance transmissions. This behavior is expected in sparse deployments dominated by long-range multipath links (∝$\:{d}^{4}$), where only the proposed protocol incorporates an adaptive energy model that mitigates this cost.
- Medium-density networks (8.16 to 25 nodes/unit area): The proposed protocol delivers significantly more packets, benefiting from its ability to maintain a larger set of active nodes, avoid premature depletion near the BS, and more effectively balance inter-cluster communication costs. K-Means-Based LEACH remains the second-best performer, while DEEC-KM consistently trails, although the gap narrows slightly as distances shorten. This trend arises because higher densities shift more transmissions into the free-space region (∝$\:{d}^{2}$), where the proposed protocol’s distance-aware energy model becomes particularly efficient.
- High-density networks (44.44 to 100 nodes/unit area): The proposed protocol continues to yield the highest packet delivery up to the 125 × 125 m^2^ configuration. However, at 100 × 100 m^2^, all three protocols converge, with packet counts of 345,050, 344,206 and 344,174 for the proposed Energy-Driven K-Means-based LEACH, traditional K-Means-based LEACH, and DEEC-KM protocols respectively. This is the only scenario in which DEEC-KM nearly matches the others, as uniformly short distances minimize the negative effects of its distance-agnostic CH selection. In very dense deployments, transmission costs become uniformly low, causing performance differences among the protocols to diminish and packet delivery to plateau.

As conclusions:

- The Energy-Driven K-Means-based LEACH protocol consistently achieves the highest number of delivered packets across all nine network areas and maintains its superiority even under very dense deployments.
- K-Means-based LEACH always outperforms DEEC-KM, except at the extreme 100 × 100 m^2^ configuration where their performance levels nearly converge.
- DEEC-KM remains the weakest overall, particularly in sparse and medium-density networks, largely due to its reliance on residual energy rather than distance for CH selection.
- At very high densities, differences between the three protocols become minimal because node-to-CH distances are uniformly short and the radio model effectively operates in the free-space region ($\:{d}^{2}$).

#### Number of better-performing packets’ files

To complement the analysis of the average number of delivered packets, we also evaluated the Number of Better-Performing Packet Files, in order to quantify how often each protocol performs better on a per-file basis. This analysis is important because the traditional K-Means-based LEACH occasionally outperforms the proposed Energy-Driven K-Means-based LEACH, especially in dense networks and in the very early phases of dense networks, where their average performance becomes very close. Since each deployment area was evaluated using 10 topologies, and for each topology 30 packet files were generated, this metric allows us to estimate how many topologies (out of 10 per area) yielded better results using the traditional protocol.

Although DEEC-KM results were obtained, they were not included in this comparison because DEEC-KM consistently produced the lowest number of delivered packets across all densities, and never surpassed the other two protocols in any of the evaluated packet files. Its inferior performance trend has already been firmly established in the earlier results, making a per-file comparison unnecessary and uninformative. Consequently, the per-topology improvement analysis focuses solely on the traditional K-Means-based LEACH and the proposed Energy-Driven K-Means-based LEACH, where meaningful differences may appear.

**Fig. 15:**
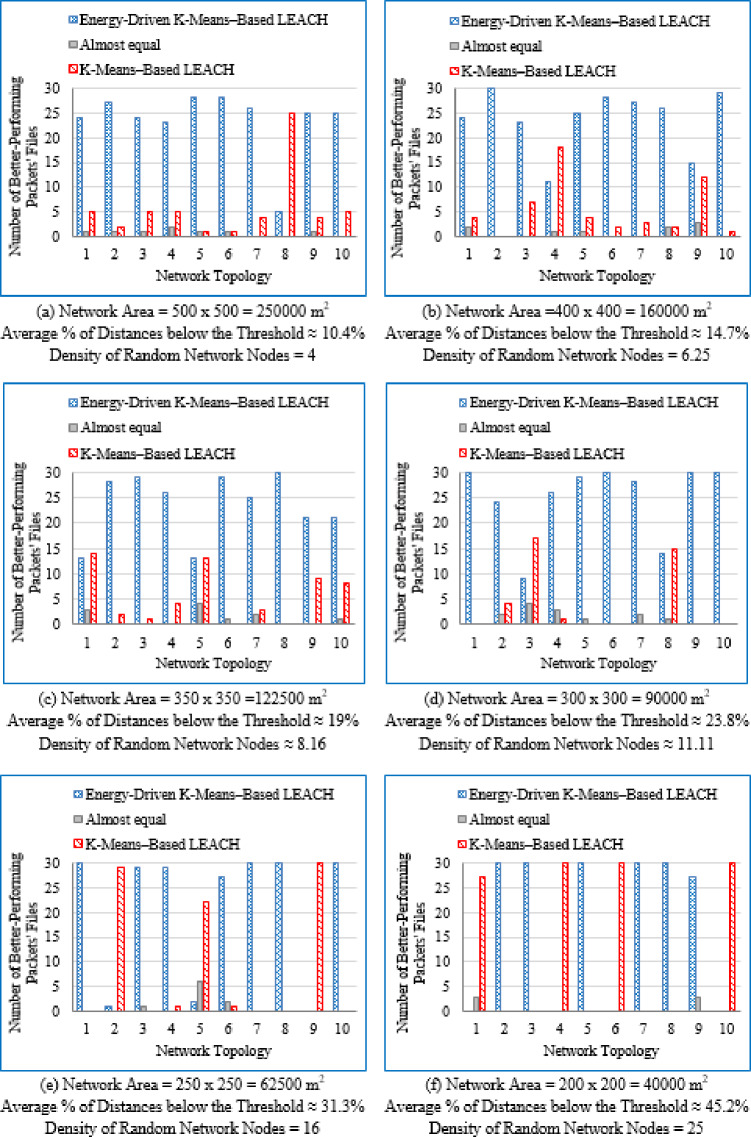

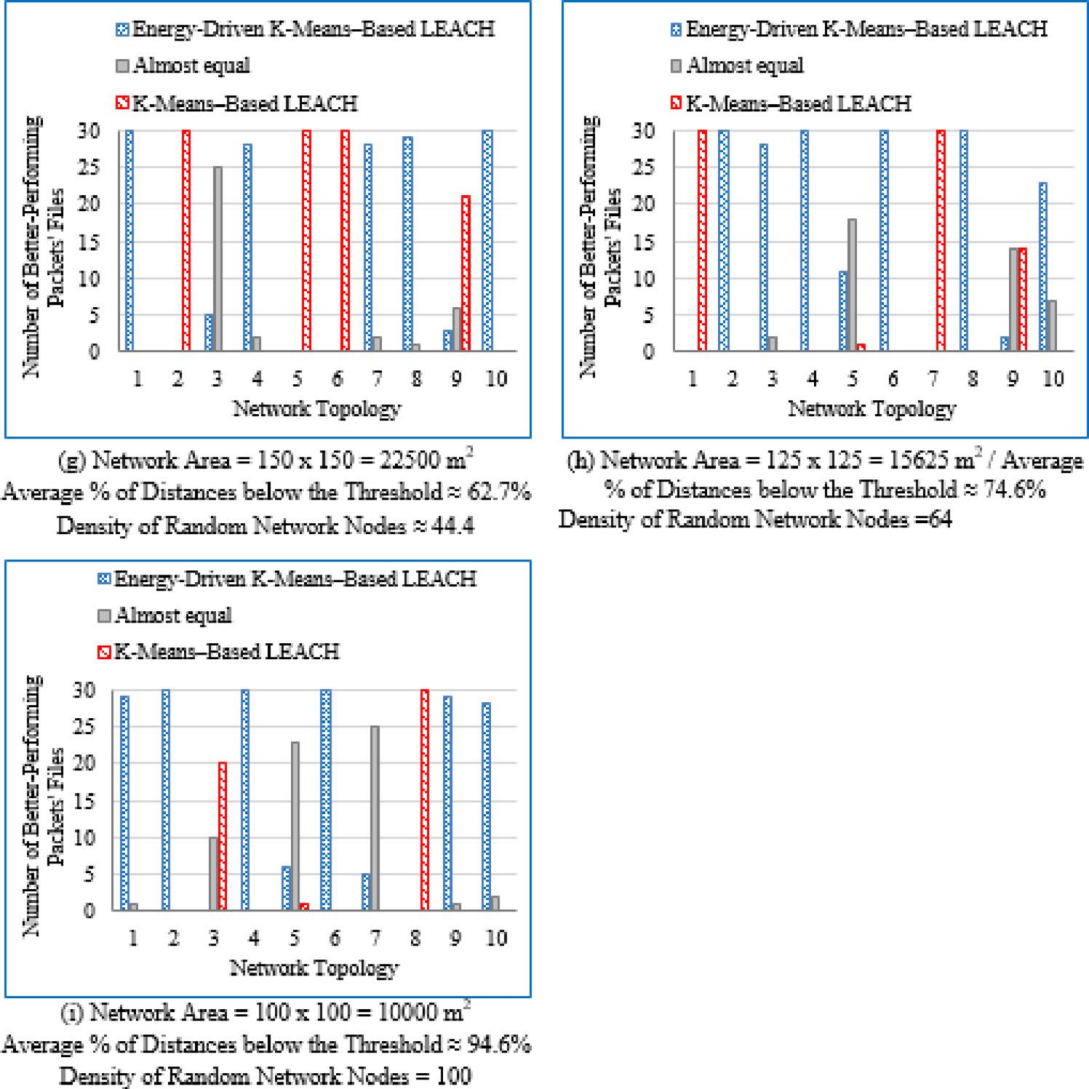
Number of better-performing packet files for the traditional K-Means-Based LEACH and the proposed Energy-Driven K-means-based LEACH routing protocols across the nine network sizes listed in Table 3, each evaluated using ten topologies and thirty randomly generated packet files per topology.

Figure 15 illustrates the number of better-performing packet files for the traditional K-Means-Based LEACH and the proposed Energy-Driven K-means-based LEACH routing protocols across the nine network sizes listed in Table 3, each evaluated using ten topologies and thirty randomly generated packet files per topology.

Table 4 Presents the domination percentage, the number of packet files needed to achieve this domination percentage, and the corresponding number of cases in which either protocol dominates. Note that 100% domination indicates that one protocol outperforms the other across all the 30 packet files, while 90–100% domination corresponds to dominance in 27 to 29 packet files, and so on.

**Table 4:** Domination percentage/corresponding number of cases.

Domination percentage	Number of packet files needed to achieve this domination percentage	Number of cases in which the energy-driven K-means-based LEACH dominates	Number of cases in which the traditional K-means—based LEACH dominates	Number of cases in which the one of the two protocols dominates
100%	30	24	10	34
90% − 100%	27–29	22	2	24
80% − 90%	24–26	12	1	13
70% − 80%	21–23	5	2	7
60% − 70%	18–20	0	3	3
				81

From Table 4, it is evident that in 81 out of 90 cases (90%), one of the two protocols demonstrates clear dominance. Specifically, the proposed Energy-Driven K-means-based LEACH dominates in 63 cases (70%), while the K-Means-Based LEACH dominates in 18 cases (20%). In the remaining 9 cases (10%), both protocols perform nearly equally, either due to a negligible difference in network death time (< 0.5%) or approximately equal performance across packet files.

**Fig. 16:**
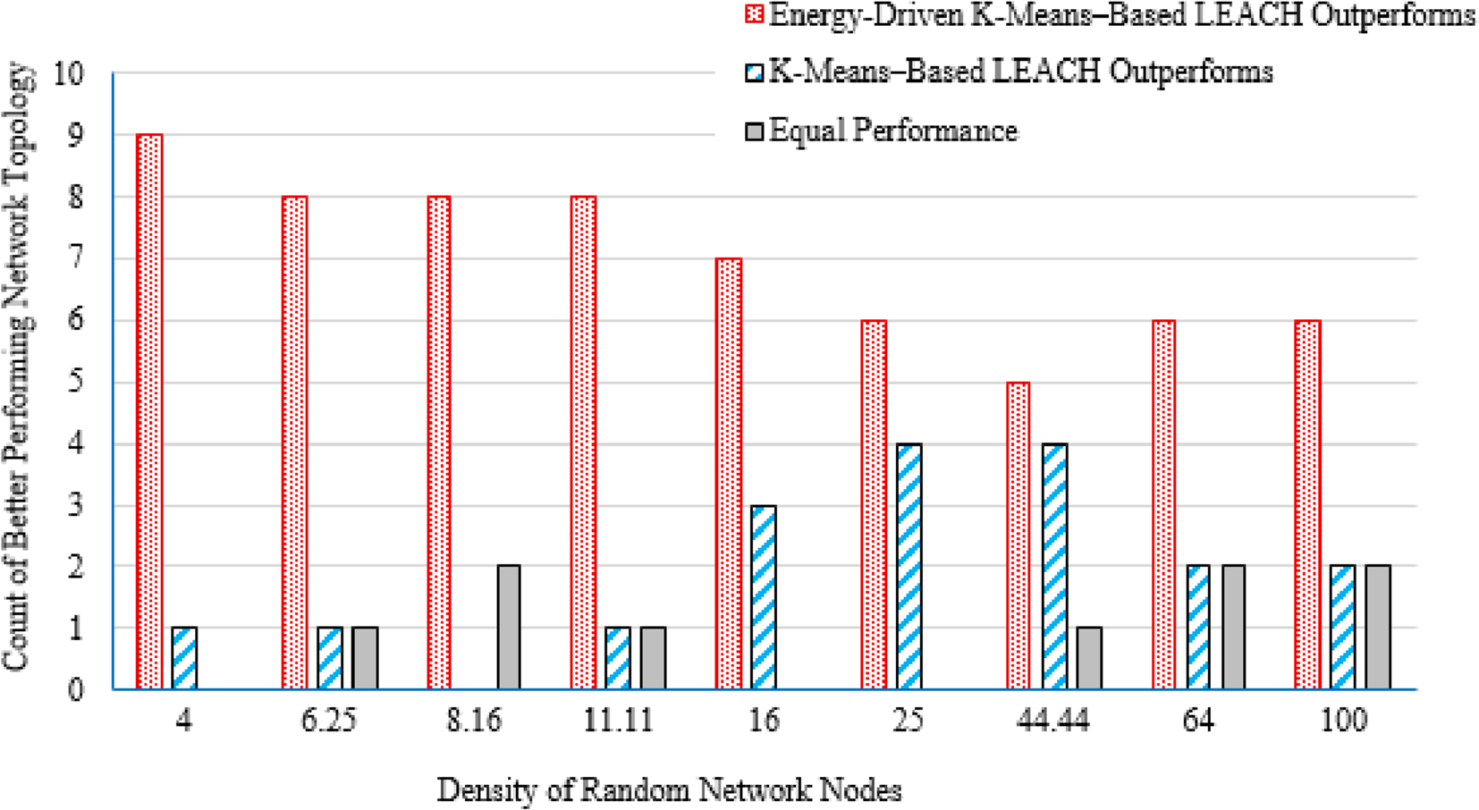
Count of better-performing network topologies using the traditional K-Means-Based LEACH and the proposed Energy-Driven K-means-based LEACH routing protocols, across the nine network densities listed in Table 3.

Figure 16 consolidates these results, illustrating the count of better-performing network topologies using both protocols across the nine node densities ($\:DRNN$). Note that the deployment area and APDN for each density are provided in Table 3 and will not be repeatedly mentioned for brevity.

From Fig. 16, the following observations can be made:

- Low-density networks (4 and 6.25 nodes/unit area): The proposed Energy-Driven K-means-based LEACH consistently outperforms the traditional K-Means-Based LEACH. Only a few scenarios favor the traditional protocol. Cases of equal performance are negligible.
- Medium-density networks (8.16 to 25 nodes/unit area): The proposed protocol demonstrates clear dominance, outperforming the traditional approach across a majority of packet files. Rare equal performance is noticed for DRNN of 8.16 and 11.11.
- High-density networks (44.44 to 100 nodes/unit area): Both protocols exhibit mixed behavior. While the Energy-Driven K-means-based LEACH frequently outperforms the traditional version, instances where the K-Means-Based LEACH performs better or where both perform equally remain relatively common.

These results highlight that the proposed Energy-Driven K-means-based LEACH protocol exhibits superior performance particularly in low-to-medium-density networks, while high-density network performance is topology-dependent.

### Summarized conclusions

The proposed Energy-Driven K-Means-based LEACH improves CH selection by replacing the standard Euclidean distance in K-means with an energy-proxy adaptive metric that switches between the $\:{d}^{2}$ and $\:{d}^{4}$ models based on inter-node distances, ensuring more energy-efficient clustering. In contrast, DEEC-KM selects CHs solely based on residual energy, ignoring transmission distance; the dominant factor in radio energy dissipation. Traditional K-Means-based LEACH relies exclusively on geometric clustering without any energy-aware mechanism, which often leads to suboptimal CH placement. The results obtained from the simulations lead to the following conclusions:

- Across all nine deployment areas, the proposed Energy-Driven K-Means-based LEACH consistently outperforms both the traditional K-Means-based LEACH and DEEC-KM in most scenarios. Its performance gains are most pronounced in low-density networks (large-scale areas with low APDN), where the distance-aware energy model yields substantial savings and significantly enhances network lifetime and energy efficiency. The traditional K-Means-based LEACH exhibits occasional early-stage advantages (small-scale areas with high APDN) and in very dense networks where all inter-node distances are short, though these cases remain limited. DEEC-KM performs the worst across all metrics, consistently reaching FND, HND, 70% ND, and LND earlier than both protocols and exhibiting the highest overall energy consumption.
- The evaluation of FND, HND, and LND time further confirms that the proposed Energy-Driven K-means-based LEACH protocol not only extends total network lifetime but also enhances early-stage stability, mid-stage energy balance, and overall endurance in nearly all but the densest scenarios.
- Regarding network lifetime enhancement, the proposed Energy-Driven K-Means-based LEACH protocol achieves up to 16.98% improvement over K-Means-based LEACH in low-density networks (large deployment areas with low APDN). The improvement gradually decreases as density increases, becoming minimal (< 2%) in very dense networks (smaller deployment areas with higher APDN). A moderate improvement of 12.5% is observed in medium-density areas. Counted negative improvements in some high-density deployments indicate that the traditional K-Means-Based LEACH protocol occasionally performs slightly better. Nevertheless, the proposed protocol maintains a predominantly positive improvement trend. No improvement is ever observed for DEEC-KM, as it consistently performs worse than both protocols.
- In terms of total energy consumption, the proposed Energy-Driven K-Means-based LEACH achieves the lowest overall power consumption across all network sizes, followed by traditional K-Means-based LEACH, while DEEC-KM remains the most energy-demanding. However, the differences among the three protocols gradually decrease in very dense networks (e.g., 100 × 100 m^2^), where their consumption curves become closely aligned. Even in these dense settings, the proposed protocol maintains a slight advantage, with its curve remaining marginally lower.
- The analysis of the average number of delivered packets and the number of better-performing packet files reinforces the same conclusion; the proposed protocol delivers more packets in almost all deployment areas. the traditional K-Means-based LEACH outperforms the proposed protocol only in a small subset of packets files, mostly in dense networks. DEEC-KM is excluded from per-file comparisons because it never performs better than the other two protocols and consistently yields the lowest packet delivery.
- In the direct comparison between the proposed Energy-Driven K-means-based LEACH protocol consistently and the traditional K-means-based LEACH routing protocols; out of the 90 evaluated cases, the proposed protocol outperforms the K-Means-Based LEACH in 63 cases, whereas the traditional K-Means-Based LEACH performs better in 18 cases; most of them occurs in very high dense networks. In the remaining 9 cases, both protocols perform nearly equivalently. This implies that in 72 out of 90 scenarios (80%), the proposed Energy-Driven K-means-based LEACH can be confidently adopted, as it either achieves superior performance (70% of the cases) or provides comparable results with no performance degradation (10% of the cases). DEEC-KM never outperforms either protocol, confirming that meaningful competition exists only between the proposed method and the traditional K-Means-based LEACH.
- In summary, the proposed Energy-Driven K-Means-based LEACH protocol is highly effective for low- to medium-density (medium- to large-scale) WSN deployments, offering substantial improvements in energy consumption, stability, and overall network lifetime. Its advantage remains present but becomes less pronounced in very dense networks, where uniformly short inter-node distances reduce the impact of distance-aware clustering.

## Conclusion and future works

This paper presented an Energy-Driven K-Means-based LEACH routing protocol for WSNs. It then evaluated it against both the traditional K-Means-based LEACH and the DEEC-KM protocols across a wide range of network densities and performance metrics. The central contribution of this work lies in introducing a novel distance metric; an analytically derived energy-proxy function; that embeds the radio energy dissipation characteristics directly into the clustering mechanism. This innovation enables CH selection and cluster formation to reflect actual communication energy cost, something not achieved by conventional Euclidean-distance clustering.

The extended comparison demonstrates that the proposed protocol achieves superior network lifetime, lower energy consumption, longer stability periods (FND, HND, 70% ND, LND), and higher packet delivery, particularly in low- and medium-density deployments where distance variation is significant.

Across all evaluated deployment areas, DEEC-KM consistently shows the weakest performance, validating its exclusion from more detailed per-file and improvement-percentage analyses.

The proposed Energy-Driven K-Means-based LEACH protocol achieves a maximum lifetime improvement of 16.98%, with strong gains across most scenarios and only marginal reductions in very dense deployments where the traditional K-Means-based LEACH occasionally performs slightly better. It is important to note, however, that although the proposed protocol delivers superior performance overall, the traditional K-Means-based LEACH still performs better in approximately 20% of the evaluated cases; mostly in very dense networks where inter-node distances are uniformly short and the benefit of energy-driven distance switching becomes minimal. These cases show that the proposed method does not universally dominate all scenarios, and that the traditional K-Means-based LEACH can remain advantageous under certain high-density conditions.

Overall, the method provides a scalable and energy-efficient clustering strategy for static or moderately sized WSNs. Its key innovation; the integration of an energy-aware distance model into K-means clustering; offers a lightweight yet impactful enhancement to centralized LEACH-based routing.

However, like most centralized clustering protocols, the Energy-Driven K-means-based LEACH is best suited for static WSN deployments and does not scale efficiently to very large networks. Moreover, its reliance on global network information and centralized computation makes it less practical for highly dynamic sensor environments.

Future work will focus on developing a machine-learning-based adaptive mechanism that selects the most suitable clustering protocol based on current network conditions, since the traditional K-means-based LEACH occasionally performs better in dense networks. The proposed Energy-Driven distance metric may also be generalized to other LEACH variants, beyond K-means-based LEACH.

Further validation against intelligent clustering approaches such as FCM, TEEN-KNN, and DL-HEED is planned. Extension to mobile and moderately dynamic WSNs or IoT deployments is also envisioned, potentially using mobility prediction or adaptive re-clustering. To improve long-term robustness, future versions may integrate real-time residual energy monitoring and adaptive CH re-selection, and explore scalable architectures such as zonal or multi-tier BS clustering. A broader evaluation including other metrics like latency is intended to broaden the analysis beyond lifetime metrics.

## Data Availability

No datasets were generated or analysed during the current study.
